# Double-activation of mitochondrial permeability transition pore opening via calcium overload and reactive oxygen species for cancer therapy

**DOI:** 10.1186/s12951-022-01392-y

**Published:** 2022-04-12

**Authors:** Ying Zhou, Shisong Jing, Sainan Liu, Xizhong Shen, Lihan Cai, Changfeng Zhu, Yicheng Zhao, Maolin Pang

**Affiliations:** 1 Center of Infectious diseases and Pathogen Biology, Key Laboratory of Organ Regeneration and Transplantation of the Ministry of Education, The First Hospital of Jilin University, Changchun, 130021 China; 2 College of Animal Science, School of Pharmacy, Jilin University, Changchun, 130022 China; 3 State Key Laboratory of Rare Earth Resource Utilization, Changchun Institute of Applied Chemistry, Chinese Academy of Science, Changchun, 130022 China; 4 University of Science and Technology of China, Hefei, 230026 People’s Republic of China; 5 Department of Gastroenterology and Hepatology, Zhongshan Hospital, Fudan University, Shanghai, 200032 China; 6 Clinical Medical College, Changchun University of Chinese Medicine, Changchun , 130117 Jilin China; 7 Shanghai Institute of Liver Diseases, Shanghai, 200001 China

**Keywords:** Ca^2+^ overload, MPTP, Hypoxia, Covalent organic frameworks, Photodynamic therapy

## Abstract

**Background:**

Calcium ions (Ca^2+^) participates in various intracellular signal cascades and especially plays a key role in pathways relevant to cancer cells. Mitochondrial metabolism stimulated by calcium overload can trigger the opening of the mitochondrial permeability transition pore (MPTP), which leads to cancer cell death.

**Methods:**

Herein, a mitochondrial pathway for tumour growth inhibition was built via the double-activation of MPTP channel. Fe^2+^ doped covalent organic frameworks (COF) was synthesised and applied as template to grow CaCO_3_ shell. Then O_2_ was storaged into Fe^2+^ doped COF, forming O_2_-FeCOF@CaCO_3_ nanocomposite. After modification with folic acid (FA), O_2_-FeCOF@CaCO_3_@FA (OFCCF) can target breast cancer cells and realize PDT/Ca^2+^ overload synergistic treatment.

**Results:**

COF can induce the production of ^1^O_2_ under 650 nm irradiation for photodynamic therapy (PDT). Low pH and hypoxia in tumour microenvironment (TME) can activate the nanocomposite to release oxygen and Ca^2+^. The released O_2_ can alleviate hypoxia in TME, thus enhancing the efficiency of COF-mediated PDT. Abundant Ca^2+^ were released and accumulated in cancer cells, resulting in Ca^2+^ overload. Notably, the reactive oxygen species (ROS) and Ca^2+^ overload ensure the sustained opening of MPTP, which leads to the change of mitochondria transmembrane potential, the release of cytochrome c (Cyt c) and the activation of caspases 3 for cancer cell apoptosis.

**Conclusion:**

This multifunctional nanosystem with TME responded abilities provided a novel strategy for innovative clinical cancer therapy.

**Graphical Abstract:**

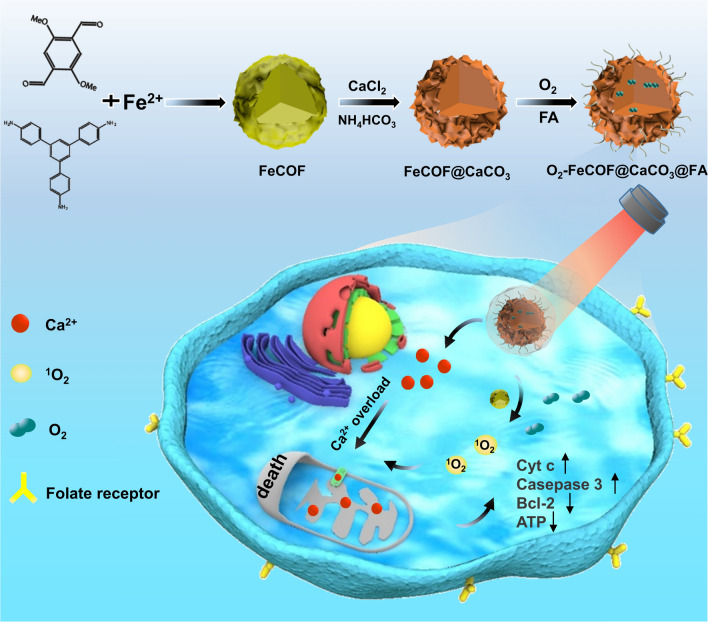

**Supplementary Information:**

The online version contains supplementary material available at 10.1186/s12951-022-01392-y.

## Introduction

Calcium signal plays an important role in various cancer progression processes such as proliferation, apoptosis and cell migration, which makes the regulation of calcium ions (Ca^2+^) in cancer cells receive increasing attention [1]. As the second messenger, Ca^2+^ has important effect on cell regulation, which can either induce cell survival or trigger apoptosis[2]. For instance, the fluctuations of Ca^2+^ content in a certain range usually promote cell proliferation and survival, whereas sustained cytosolic calcium induced Ca^2+^ overload can lead to cell apoptosis [3, 4]. Especially, tumor cells are more sensitive to Ca^2+^ overload than normal cells [5–7]. Therefore, it is a promising strategy for triggering intracellular Ca^2+^ overload to realise antitumor therapy.

As one of the intracellular Ca^2+^ pools, mitochondrial uptakes Ca^2+^ depending on the changes of transmembrane potential [8]. Mitochondrial permeability transition pore (MPTP), known as the mitochondrial megachannel, is a non-selective channel across the inner and outer layers of the mitochondrial membrane [9]. It has been reported that the continuous opening of MPTP is the direct cause of cancer cell apoptosis [10]. At physiological levels, Ca^2+^ can activate the transient opening of MPTP, allowing protons or positive ions to enter the mitochondrial matrix to prevent excessive accumulation in the mitochondrial intermembrane space. However, MPTP can be activated to open continuously due to high [Ca^2+^]m, high level of reactive oxygen species (ROS) and adenosine triphosphate (ATP) depletion, resulting in mitochondrial and cancer cells dysfunction [11–15]. Obviously, Ca^2+^ is the most important regulator and inductor for MPTP opening [16, 17]. CaCO_3_, a calcium-based biomineralized nanomaterial responding to the tumor acidic microenvironment with pH level as low as 6.2, has excellent degradability and good biocompatibility. As a natural Ca^2+^ reservoir, CaCO_3_ can provide sufficient Ca^2+^ source during the process of tumor treatment [18–23]. Moreover, when mitochondrial Ca^2+^ overload occurs, ROS can promote Ca^2+^ to stimulate sustained MPTP opening [24].

Photodynamic therapy (PDT) is a photosensitized reaction with biological effects, which involves oxygen molecules. The process is that the photosensitizer (PSs) absorbed by the tissue is excited by laser irradiation of a specific wavelength, and the excited PSs transfer energy to the surrounding oxygen to produce singlet oxygen (^1^O_2_) with strong activity. ^1^O_2_ reacts with adjacent biological macromolecules to produce cytotoxicity, resulting in cell damage and even death [25–27]. Compared with conventional treatments such as surgery, chemotherapy and radiotherapy, PDT has the advantages of less trauma, low toxicity and high selectivity [28–30]. However, PDT-mediated continuous oxygen consumption would aggravate hypoxia of cancer cells, which further blunts the therapeutic effect of PDT [31, 32]. Therefore, oxygen storage materials, including hemoglobin [33], perfluorocarbon [34] and metal organic framework (MOF) [35], are used to alleviate hypoxia in tumors.

As an emerging type of porous crystal material, covalent organic frameworks (COF) have been studied for cancer treatment in addition to their conventional applications in energy storage, catalysis, sensing and separation [36], Owe to their unique structure and characteristics such as porosity, stability and biocompatibility, COF has been widely applied for drug delivery, photodynamic therapy and photothermal therapy [37–40], Tan and his colleagues, for example, reported a porphyrin-COF for photodynamic therapy of tumors. Under near-infrared irradiation, COF nanoparticles produced abundant ROS to induce cancer cell apoptosis result in their crystalline network structure [41], Li and his colleagues synthesized a porous 8-hydroxyquinoline functionalized organic covalent framework (COF-HQ) with pH-sensitive tumor microenvironment for 5-FU loading, achieving efficient drug delivery and anti-tumor effects [42]. To the best of our knowledge, the use of COF as a smart carrier for oxygen molecules to achieve tumor-targeted transport has not been reported.

**Scheme 1:**
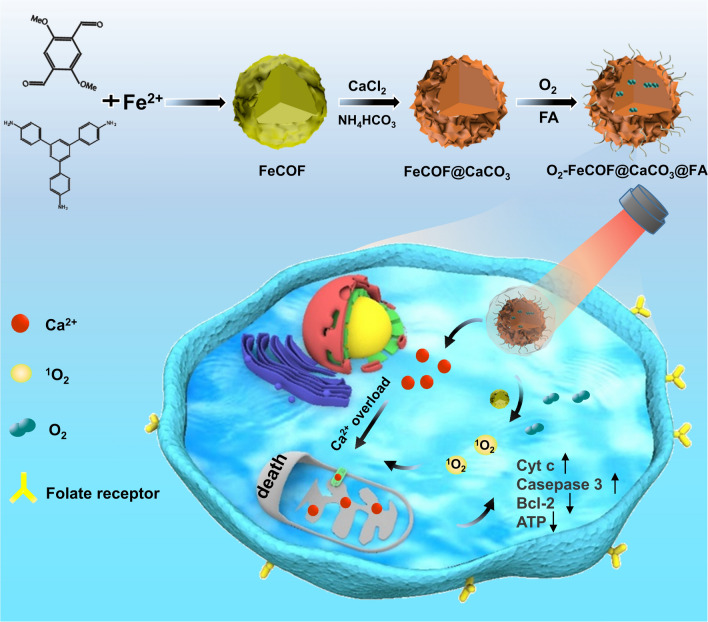
The synthetic route of OFCCF and OFCCF-based Ca^2+^ release and production of ^1^O_2_ activation of MPTP opening inhibits tumor growth strategy

Herein, we synthesized Fe^2+^-doped COF by Schiff base reaction at room temperature. Then FeCOF was used as a template for the growth of FeCOF@CaCO_3_ by gas diffusion method. And folic acid (FA) was modified on the surface of FeCOF@CaCO_3_ to form FeCOF@CaCO_3_@FA (FCCF) for achieving targeted therapy against breast cancer. FCCF possessed highly efficient oxygen-carrying capacity owing to the affinity of doped Fe^2+^ with oxygen. Upon internalization into breast cancer cells, O_2_-FeCOF@CaCO_3_@FA (OFCCF) nanoparticles were disassembled, releasing O_2_ and Ca^2+^. COF, as the photosensitizer, was used to produce ROS under light exposure*.* The released O_2_ could enhance COF-mediated PDT in hypoxic tumour microenvironment (TME). Moreover, the coated CaCO_3_ layer exhibited excellent pH-dependent dissociation behavior, causing rapid release of Ca^2+^ under the acidic conditions. Large amounts of Ca^2+^ could accumulate in mitochondria, leading to the disruption of Ca^2+^ homeostasis and mitochondria dysfunction. Therefore, the high levels of ROS and intracellular accumulation of Ca^2+^ induced the continuous opening of MPTP, causing an influx of Ca^2+^ to kill cancer cells. Besides, pathological and biochemical tests confirmed that OFCCF nanoparticles could avoid the disturbance of systemic toxicity (Scheme 1).

## Results

### Synthesis and characterization of O_2_-FeCOF@CaCO_3_@FA NPs

**Fig. 1:**
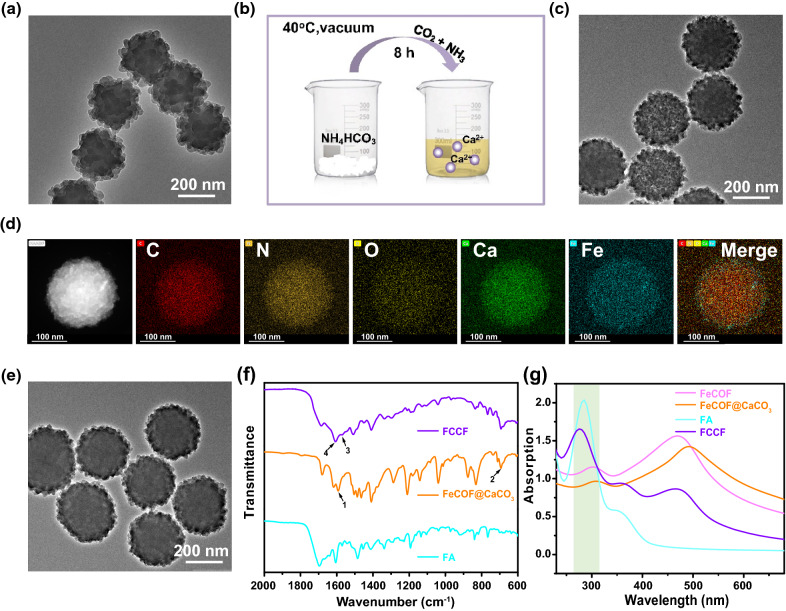
**a** TEM image of FeCOF. **b** Schematic diagram of synthesis method of FeCOF@CaCO_3_. **c** TEM image and (**d**) elemental mapping of FeCOF@CaCO_3_. **e** TEM image of FCCF. **f** FTIR spectra of FA, FeCOF@CaCO_3_ and FCCF. **g** UV–vis absorption spectra of FeCOF, FeCOF@CaCO_3_, FA and FCCF

The well-dispersed spherical FeCOF was simply synthesized by using acetic acid as catalyst at room temperature (Fig. 1a and Additional file 1: Figure S1). FeCOF@CaCO_3_ was prepared via gas diffusion method. As illustrated in Fig. 1b, in a closed vacuum, the CO_2_ and NH_3_ gases produced by the natural decomposition of NH_4_HCO_3_ would continuously diffuse into the mixed solution containing Ca^2+^. Simultaneously CO_3_^2−^ was provided into the alkaline solution to trigger the formation of CaCO_3_ [43]. Thin nanosheets of CaCO_3_ could be observed on the surface of FeCOF by transmission electron microscopy (TEM, Fig. 1c) and scanning electron microscopy (SEM, Additional file 1: Figure S2). Elemental mapping analysis showed that C, O, N, Fe and Ca elements of FCCF nanoparticles were homogeneously distributed (Fig. 1d). X-ray photoelectron spectroscopy (XPS) was used to analyse Ca element signal of FeCOF@CaCO_3_. The two peaks located at 347.2 eV and 350.7 eV for 2_p1/2_ and 2_p3/2_, respectively, were considered as the characteristic peaks of CaCO_3_ (Fig. S3) [44]. Powder X*-*ray diffraction (PXRD) patterns have confirmed the excellent crystallinity of FeCOF and FeCOF@CaCO_3_ (Additional file 1: Figure S4) [37, 45]. The surface area and pore volume of FeCOF and FeCOF@CaCO_3_ were 1380.170 m^2^/g, 1.292 cc/g and 373.731 m^2^/g, 0.305 cc/g. Compared with FeCOF, FeCOF@CaCO_3_ showed decreased Brunauer–Emmett–Teller (BET) surface area and pore volume, confirming the successful coating of CaCO_3_ (Additional file 1: Figure S5). Then FA was modified on the surface of FeCOF@CaCO_3_ to obtain the final FeCOF@CaCO_3_@FA (FCCF) nanoparticles (Fig. 1e). In the FTIR spectra of FeCOF@CaCO_3_, the peak at 1592 cm^−1^ (peak 1) was ascribed to the vibration of -NH_2_ and the characteristic peak at 712 cm^−1^ (peak 2) belonged to carbonate. In the FTIR spectra of FCCF, the characteristic peaks at ~ 1573 (peak 3) and 1647 cm^−1^ (peak 4) have been characterized as the stretching vibration mode of secondary amide bonds (C = O-NH), further demonstrating the successful synthesis of FCCF (Fig. 1f), further demonstrating the successful synthesis of FCCF [46, 47]. UV–visible (UV-vis) absorption spectrum of FCCF showed that there was a broad absorption between 400 and 700 nm, which was similar to the absorption of FeCOF nanoparticles. Meanwhile, the strong absorbance peak centered at 281 nm of FCCF validated the integration of FA into the nanosystem (Fig. 1g). TEM and Dynamic light scattering (DLS) measurements showed that the mean size of FCCF was about 230 nm (Additional file 1: Figure S6 and Table S1), slightly larger than that of FeCOF and FeCOF@CaCO_3_ nanoparticles. In addition, the successful synthesis of FCCF could also be proved by the zeta potential changes (Additional file 1: Figure S7). The post-modification of FA could enhance the bio-compatible and tumor targeting abilites of FCCF. As shown in Additional file 1: Figure S8, FCCF composite were uniformly dispersed in water, phosphate buffered saline (PBS) and cell culture medium (containing 10% serum) for 7 days without any aggregation at room temperature. The morphology of the nanocomposite had no change even after incubation in water for a week. These results verified the good stability of FCCF for potential clinical applications.

### In vitro release study

**Fig. 2:**
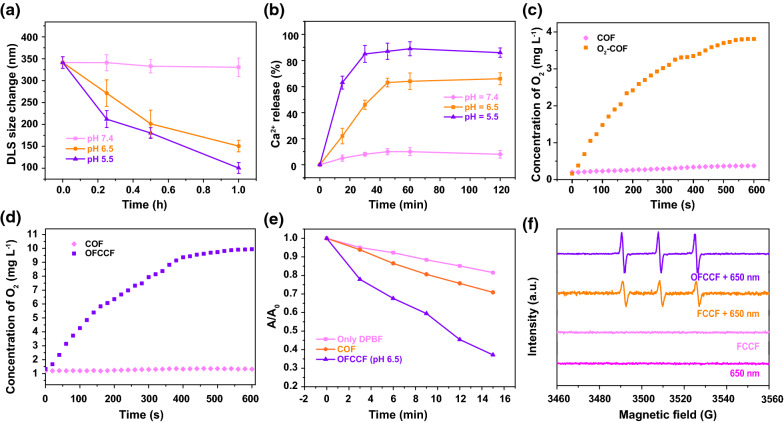
**a** Time-dependent DLS-measured size changes of FCCF under different pH conditions (pH 7.4, 6.5 and 5.5). **b** Time-dependent release of Ca^2+^ from the FCCF dispersed in pH 7.4, 6.5 and 5.5 buffers. **c** Time-dependent dissolved oxygen generation induced by O_2_-COF in deoxidized PBS. **d** Time-dependent dissolved oxygen generation induced by OFCCF in deoxidized PBS (pH 6.5). **e** UV–Vis absorption of DPBF after 650 nm laser irradiation with H_2_O, COF (100 μg mL^−1^) and OFCCF (100 μg mL^−1^, pH 6.5). **f** ESR spectra of FCCF and OFCCF under 650 nm laser irradiation (0.72 W cm^−2^, 10 min)

In order to verify TME-activated bio-decomposition abilities of FCCF nanoparticles, various measurements were conducted. FCCF and FeCOF nanoparticles were dispersed in PBS with different pH to observe the morphology changes at different time points. As shown in Additional file 1: Figure S9 and S10, FCCF nanoparticles showed no change on size and structure when dispersed in PBS at pH 7.4. In contrast, FCCF nanoparticles dissociated in PBS with low pH (6.5 and 5.5), resulting in the nanosheet like morphology of the surface was largely lost after 1 h. Meanwhile, the hydrodynamic sizes of nanoparticles for time-dependent changes were monitored by DLS at different pH values. The hydrated particle of FCCF nanoparticles at pH 6.5 and 5.5 for 1 h were around 180 nm and 100 nm, respectively (Fig. 2a). Then, the time-dependent release profiles of Ca^2+^ were further assessed in buffers with various pH values. As shown in Fig. 2b, the low pH condition (5.5) led to a sustained release of Ca^2+^, with 89.2% Ca^2+^ being released from FeCOF carrier. However, only about 66.4% Ca^2+^ was released after being incubated at pH 6.5. The above results strongly proved that FCCF exhibited promising pH-responsive Ca^2+^ release ability.

The release of Ca^2+^ further facilitated oxygen diffusion to meet the increased oxygen demand in hypoxic tumor. O_2_ releasing ability of OFCCF and O_2_-COF in deoxidized PBS buffer was illustrated in Fig. 2c, d. The storaged O_2_ of OFCCF and O_2_-COF were released under a hypoxic environment by passive transportation. However, the released O_2_ from OFCCF was approximately twofold as that from O_2_-COF, which was attributed to the affinity of Fe^2+^ with O_2_. Moreover, the bubbles produce in the OFCCF solution also demonstrated the specific oxygen release behavior of Additional file 1: Figure S11. The ROS generation ability of OFCCF was investigated by 1,3-diphenylisobenzofuran (DPBF). Compared with free DPBF, COF showed effective time-dependent ROS production under 650 nm (0.72 W cm^−2^) laser irradiation, which was attributed to its strong absorption (Fig. 1g). Moreover, the released O_2_ could enhance the production of ROS (Fig. 2e). Next, electron spin resonance (ESR) with 2,2,6,6-tetramethyl-4-piperidinol (TEMP) as singlet ^1^O_2_ trapping agent was used for detecting ^1^O_2_ generation. ^1^O_2_ signal (1:1:1) was observed for OFCCF under 650 nm laser irradiation, which was stronger than that of FCCF (Fig. 2f). Overall, OFCCF with excellent ^1^O_2_ generation ability could effectively overcome tumor hypoxia and enhance PDT effect for breast cancer.

### Cancer cell death induced by calcium overload

**Fig. 3:**
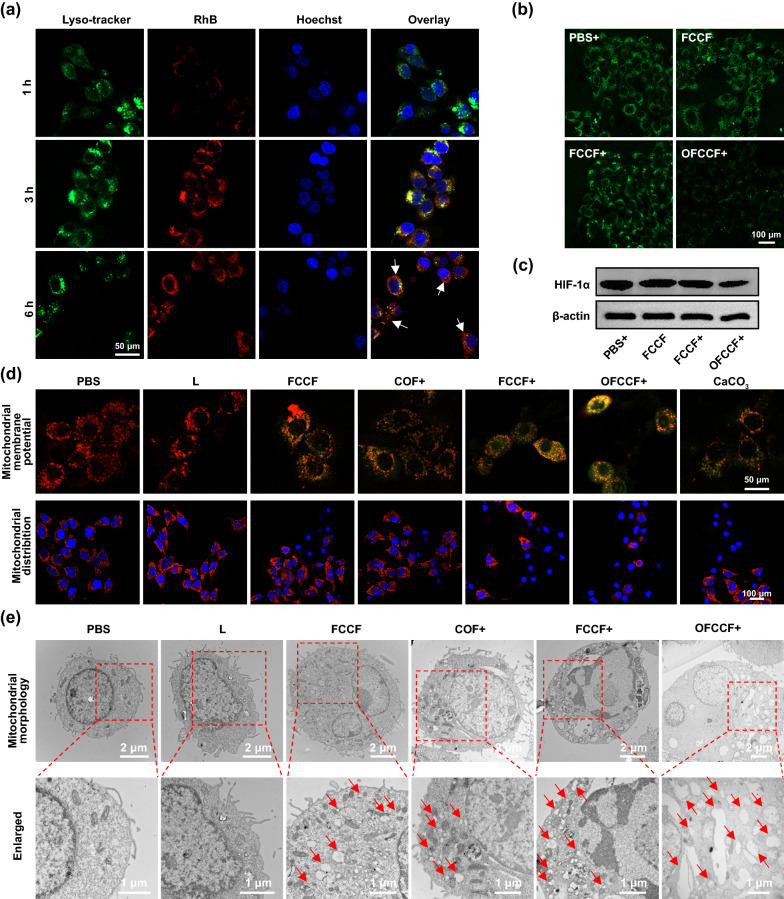
**a** The images of 4T1 cells incubated with FCCF recorded at different time points. **b** [Ru(dpp)_3_]Cl_2_ was used as probe to detect O_2_ generation after treatment with: (1) PBS + , (2) FCCF, (3) FCCF + and (4) OFCCF + . **c** Western blot analysis of HIF-1α expression of 4T1 cells. **d** Mitochondrial distribution and mitochondrial membrane potential images of 4T1 cells after different treatments. **e** Bio-TEM images of 4T1 cells after different treatments. Red arrows exhibit the location of destructed mitochondria in 4T1 cells

Next, the cellular internalization of rhodamine B (RhB)-labelled FCCF nanoparticles was examined in murine 4T1 breast cancer cells. Figure 3a indicated the time-dependent internalization process of FCCF nanoparticles, as evidenced by the colocalization of red fluorescence for RhB-labelled FCCF nanoparticles and the green fluorescence for LysoTracker. These results improved that FCCF nanoparticles could be effectively uptaken by 4T1 cells, which was beneficial to killing tumor cells. Within acidic lysosomes, OFCCF nanoparticles could release Ca^2+^ rapidly, which led to a direct increase of osmotic pressure and influx of Cl^−^ and H_2_O molecules to result in proton spongeeffect [21, 46, 47]. Simultaneously, under 650 nm laser irradiation, a large number of ^1^O_2_ were produced by the nanoparticles, causing the destruction of lysosome membrane structure to favor the endosomal escape of nanoparticles. Then, intracellular Ca^2+^ concentration was monitored using the calcium indicator dye Fluo-4. Weak green fluorescence was observed in PBS group and L group of 4T1 cells. In contrast, compared with other groups, the intracellular Ca^2+^ concentration in the OFCCF + group was highest (Additional file 1: Figure S12). In addition, mitochondrial Ca^2+^ concentrations were quantified using the calcium indicator dye Rhod*-*2. The FCCF, COF + and CaCO_3_ groups exhibited weak red luminescence, while the group treated with OFCCF + exhibited strong intracellular luminescence, indicating Ca^2+^ influx of 4T1 cells could be better activated in the presence of ROS (Additional file 1: Figure S13). Via Calcium Colorimetric assay, the OFCCF + displayed the highest intracellular Ca^2+^ concentration as 19.4 μg/mL, indicating an obvious Ca^2+^ overloading (> 3.2 μg/mL in PBS group) (Additional file 1: Figure S14). The release of Ca^2+^ and the hypoxic tumor microenvironment would trigger the free diffusion of oxygen by passive-transport. The O_2_ probe [Ru(dpp)_3_]Cl_2_ (RDPP) which is prone to luminescence quenching by oxygen was used to monitor cellular O_2_-evolving. As shown in Fig. 3b, the green fluorescence intensity of OFCCF group decreased obviously under hypoxic conditions, while the green fluorescence was observed for L treated and FCCF treated groups. Then, quantitative analysis of dynamic changes of intracellular oxygen via calculating average intensity by ImageJ software. The green fluorescence intensity of 4T1 cells treated with OFCCF + was 22%, which was about three times lower than the other three groups (Additional file 1: Figure S15). Meanwhile, as the expression of HIF*-*1α protein is upregulated under a hypoxic condition, the degree of hypoxia can be further assessed according to the level of HIF*-*1α. The OFCCF treated 4T1 cells exhibited a low expression of HIF*-*1α by western blotting (WB) analysis (Fig. 3c and Additional file 1: Figure S16). These results suggested that OFCCF could release O_2_ to alleviate the hypoxic state of tumor microenvironment. The enhance of ROS generation by OFCCF-mediated O_2_ was proved by intracellular ^1^O_2_ test with 2ʹ,7ʹ-dichlorodihydrofluorescein diacetate (DCFH-DA) fluorescence as a probe. As shown in Additional file 1: Figure S17, a comparative intensity of green fluorescence was observed under hypoxia and normoxia conditions, suggesting the key role of oxygen release in PDT.

Ca^2+^ overload and ^1^O_2_ can cause the conformational changes of MPTP structural proteins, which allows substances with a molecular weight greater than 1500 to pass through the inner mitochondrial membrane (IMM) by non-selectively way. The entered substances result in the collapse of mitochondrial membrane potential (MMP), the uncoupling of oxidative phosphorylation process and the disturbance of ATP production, consequently causing mitochondria function impairment [16]. Therefore, we used commercial Calcein-AM or Calcein-AM + CoCl_2_ dye as a fluorescence indicator to evaluate whether OFCCF was able to induce the continuous activation of MPTP. Upon treatment with FCCF or COF + , green fluorescence was observed. In contrast, no green fluorescence was detected for OFCCF group. However, upon receiving simultaneous exposure of OFCCF + and 5 µM uncoupling agent (CCCP), the 4T1 cells exhibited green fluorescence signal (Additional file 1: Figure S18), suggesting the critical role of Ca^2+^ overloading to activate MPTP opening. Next, to reveal the degree of mitochondrial damage induced by enhanced mitochondrial Ca^2+^ overload, the mitochondrial membrane potential of 4T1 cells was evaluated using commercial JC-1 dye. The OFCCF + group showed strong green fluorescence signal, in marked contrast to the strong red fluorescence observed in other groups. Subsequently, intracellular mitochondrial distribution was detected by staining with Mito Tracker^®^ Red CMXRos. As expected, the fewest mitochondria damage were detected in the OFCCF + group (Fig. 3d and Additional file 1: Figure S19). Finally, biological transmission electron microscopy (Bio-TEM) was used to visualize changes in their mitochondria (Fig. 3e). The 4T1 cells treated with FCCF, COF + or FCCF + exhibited only mild mitochondrial destruction, whereas OFCCF + caused the most obvious mitochondrial destruction, with visible swelling and cavitation of mitochondria. These results confirm that OFCCF + can cause severe mitochondrial damage through mitochondrial Ca^2+^ overload. In addition, the intracellular ATP content treated with OFCCF + decreased significantly in comparison to other five groups, which was partially produced via the oxidative phosphorylation inside the mitochondria (Additional file 1: Figure S20). All of the results revealed that the increased intracellular Ca^2+^ and production of ^1^O_2_ induced by OFCCF + could cause mitochondrial dysfunction.

**Fig. 4:**
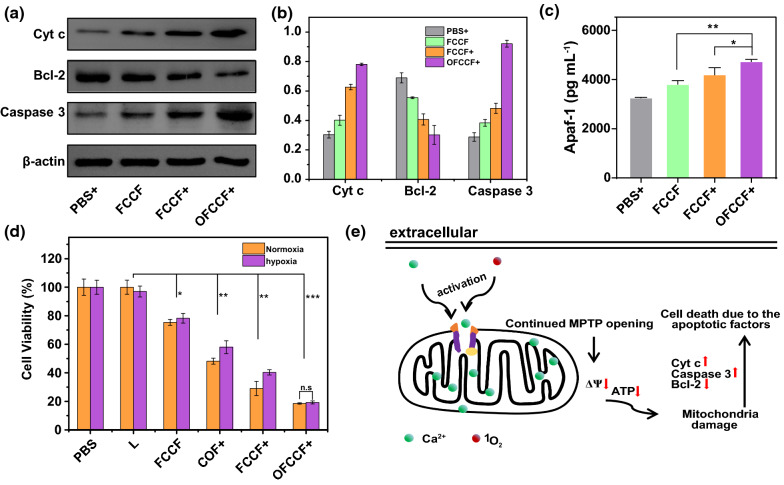
**a** Western blot analysis Cyt c, Bcl-2 and Caspase 3 expression of 4T1 cells. **b** Quantitative analysis of Cyt c, Bcl-2 and Caspase 3 expression. **c** Apaf-1 level of cell supernatant after various treatments. *P* values were calculated by one-way analysis (*p < 0.05, **p < 0.01, ***p < 0.001). Data were represented as mean ± SD (n = 6). **d** Cytotoxicity profiles of 4T1 cells treated with: (1) PBS, (2) L, (3) FCCF, (4) COF + , (5) FCCF + and (6) OFCCF + . *P* values were calculated by one-way analysis (*p < 0.05, **p < 0.01, ***p < 0.001). Data were represented as mean ± SD (n = 4). **e** Schematic diagram of mitochondrial damage induced by bidirectional activation of MPTP opening by Ca^2+^ and ^1^O_2_

The collapse of MMP induces a series of pathological changes in mitochondria, resulting in the release of Cyt c from mitochondrial matrix into cytoplasm. And Cyt c and apoptotic protein activator-1 (Apaf-1) can form a composite to trigger cell apoptosis [48]. To confirm this principle, the expression of apoptosis-related proteins was investigated by western blotting*.* As illustrated in the Fig. 4a, the expression levels of Cyt c and caspase 3 increased after the treatment of OFCCF + in comparison to the PBS, FCCF and FCCF + groups. By contrast, the protein levels of Bcl-2 in 4T1 cells were markedly down-regulated after OFCCF + treatment. Detailed quantitative results of WB were gathered in Fig. 4b. In addition, the release of Apaf-1 from the supernatant of 4T1 cells was measured by enzyme-linked immunosorbent assay (ELISA). As shown in Fig. 4c, the OFCCF + treatment elevated the release of Apaf-1 effectively. The above results indicated that mitochondrial-mediated apoptotic pathway was activated after the mitochondrial Ca^2+^ overload and production of ^1^O_2_.

Then, the cytotoxic effect of COF and FCCF nanoparticles towards L929 fibroblast cells and 4T1 cells was evaluated using the standard methyl thiazolyl tetrazolium assay (MTT). L929 cells were treated with various concentrations of COF and FCCF nanoparticles within 24 and 48 h, the survival rate maintained above 80%. The proliferation ability of L929 cells treated with OFCC + and OFCCF + decreased slightly (Additional file 1: Figure S21). These results demonstrated the biosafety and biocompatibility of the nanoparticles. To further investigate the therapeutic effect of Ca^2+^ overload/PDT, the anti-cancer effect of OFCCF under hypoxic and normoxic environment was tested. Upon 650 nm laser irradiation, the cytotoxicity of FCCF + under normoxic environment was greatly enhanced than that of FCCF + under the hypoxic environment. Notably, the hypoxia condition had significant effect on the cancer cell killing effect of FCCF + -treated group. While the survival rate of OFCCF + treated group was almost the same as the group under hypoxia condition owing to the oxygen-carrying properties of FeCOF (Fig. 4d). Moreover, from the live/dead cell staining test, we observed that most cells remained alive after the treatment of PBS and L. By contrast, the OFCCF + treated group showed a large number of dead cells in the Additional file 1: Figure S22. This result was consistent with the flow experimental results (Additional file 1: Figure S23). Collectively, all above results reveal that OFCCF is a promising candidate to induce cancer cell death through Ca^2+^ overload and ^1^O_2_ co-activating MPTP opening (Fig. 4e).

### In vivo therapeutic effect investigation

**Fig. 5:**
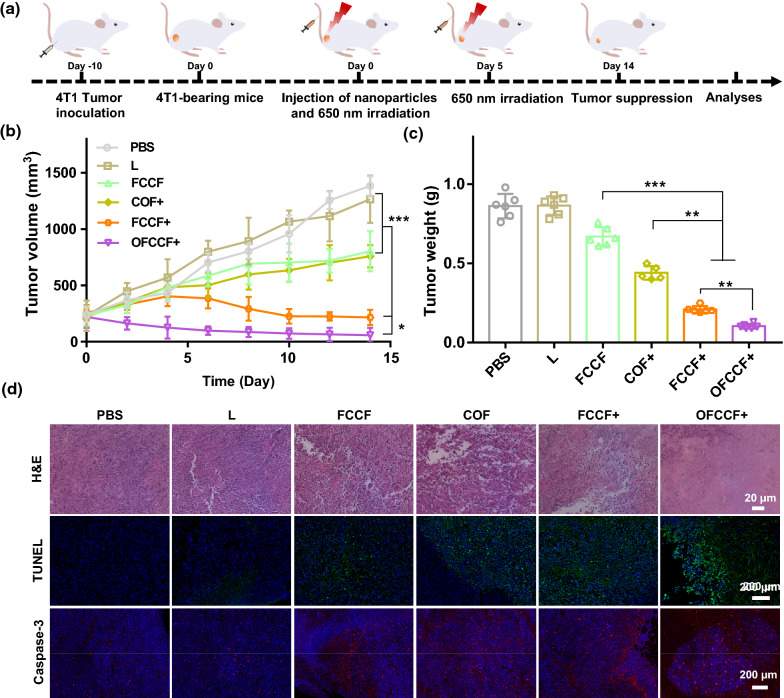
**a** Schematic description of the establishment of 4T1 tumor model and therapeutic outcome. **b** The relative tumor volume of mice in different groups after treatment. *P* values were calculated by one-way analysis (*p < 0.05, **p < 0.01, ***p < 0.001). Data were represented as mean ± SD (n = 6). **c** Tumor weight in different groups after treatment. *P* values were calculated by one-way analysis (*p < 0.05, **p < 0.01, ***p < 0.001). Data were represented as mean ± SD (n = 6). **d** H&E, TUNEL and Caspase 3 staining of tumor collected from mice after various treatments

The in vivo therapeutic efficacy of OFCCF was further examined by a 4T1 breast tumor model. The tumor-bearing mice were randomly divided into six groups for the different treatments: (1) PBS, (2) 650 nm laser irradiation (L), (3) FCCF, (4) COF + , (5) FCCF + and (6) OFCCF + . After intravenously injection with nanocomposites (100 μg mL^−1^) for 12 h, the mice were treated with 650 nm laser irradiation (0.72 W cm^−2^) for 5 min (Fig. 5a). The obtained nanocomposites had no side effect on the body weight of mice, suggesting the good biosafety of the nanocomposites (Additional file 1: Figure S24). Tumor suppression assessments showed a considerable suppression effect on the tumors of FCCF + group and OFCCF + group in comparison with other four groups*.* (Fig. 5b)*.* This result was consistent with the pictures of mice tumors and tumor weight (Fig. 5c and Additional file 1: Figure S25). Furthermore, hematoxylin and eosin (H&E) staining results were shown in Fig. 5d, obvious cell necrosis and apoptosis could be observed in OFCCF + group. From the TUNEL staining results, the green signal represented that cells apoptosis was appeared after the treatment of OFCCF + . Meanwhile, the typical morphology of apoptotic cells was detected in caspase 3 staining assay. Finally, to better assess the effect of this synergistic therapy, we next examined the lung metastasis of 4T1 tumor-bearing mice after various treatments. H&E staining and imaging of lung sections confirmed that OFCCF + effectively suppressed tumor metastasis (Additional file 1: Figure S26).

**Fig. 6:**
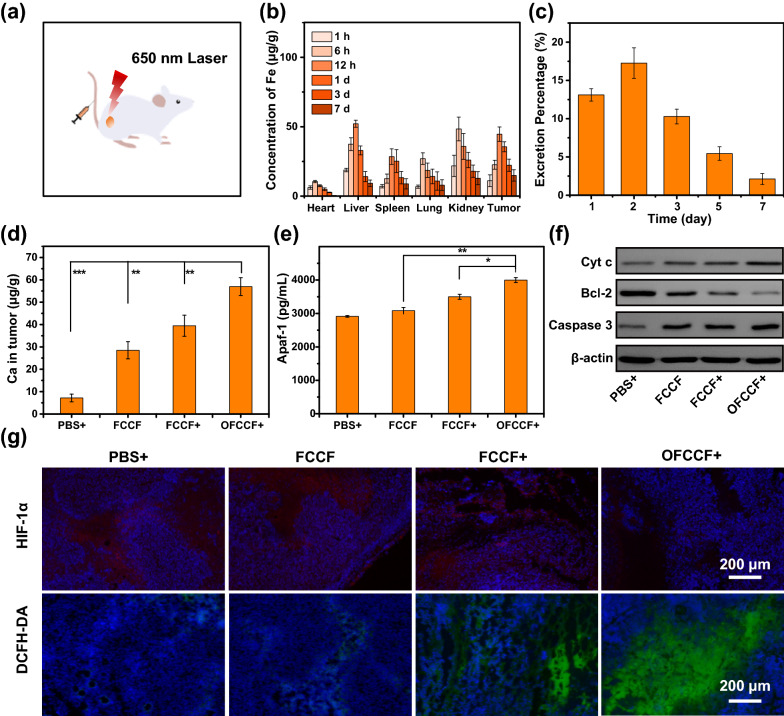
**a** Schematic illustration of OFCCF-based Ca^2+^ release and production ^1^O_2_ activation of MPTP opening inhibits tumor growth. **b** The biodistribution of Fe in main tissues and tumor (**c**) and feces in different times after intravenously injecting with OFCCF into 4T1-tumor-bearing. Data were represented as mean ± SD (n = 3). **d** The biodistribution of Ca in tumor after various treatments at 12 h intravenously injecting into 4T1-tumor-bearing. Data were represented as mean ± SD (n = 3). **e** Apaf-1 level of tumor supernatant after various treatments. *P* values were calculated by one-way analysis (*p < 0.05, **p < 0.01, ***p < 0.001). Data were represented as mean ± SD (n = 3). **f** Western blot analysis Cyt c, Bcl-2 and Caspase 3 expression of tumor. **g** HIF-1α and DCFH-DA staining of tumor collected from mice after various treatments

Moreover, the therapeutic efficiency of OFCCF + was further evaluated (Fig. 6a). To study the bio-distribution of FCCF,FCCF was injected intravenously into 4T1-tumor-bearing mice and Fe content was tracked by inductively coupled plasma mass spectrometry (ICP-MS). As shown in Fig. 6b, nanoparticles preferentially accumulated at the tumor site due to the specific binding of FA with breast cancer folate receptors, showing the highest content at 12 h after injection. Apart from tumors, the nanoparticles were also accumulated in liver and kidney. As shown in Fig. 6c, the content of FCCF nanoparticles in liver and kidney decreased gradually, confirming that they could be metabolized from the body through the renal-urinary clearance system within one week. The tumor accumulation and clearance performance of FCCF provides enormous potential for effective and safe cancer treatment. Furthermore, the bio-degradation performance of nanoparticles triggered by TME was further studied. After the PBS, FCCF and OFCCF was injected intravenously into 4T1-tumor-bearing mice for 12 h, respectively, the Ca^2+^ released from nanoparticles accumulated in tumor owing to the acidic condition of TME. As we expected, the Ca^2+^ content in the tumors significantly increased after being treated with OFCCF + in comparison to FCCF and FCCF + groups. It is further revealed that the production of ROS could promote the aggregation of Ca^2+^, resulting in the apoptosis caused by Ca^2+^ overload (Fig. 6d). Next, the possible mechanism of Ca^2+^ overload-induced apoptosis was explored by ELISA and western blotting to determine the content of apoptosis-related proteins of tumours*.* Once Cyt c is released from the mitochondria, it can couple with Apaf-1 to form the apoptosome. As illustrated in Fig. 6e, the upregulation of Apaf-1 confirmed the formation of apoptosome. Besides, the expression levels of Cyt c, Bcl-2 and caspase 3 were further detected by western blotting. As illustrated in the Fig. 6f, compared with the control groups (PBS, FCCF and FCCF +), Cyt c and caspase 3 expressions levels increased significantly but the protein levels of Bcl-2 decreased after OFCCF + treatment. The quantitative analysis of western blotting was shown in Additional file 1: Figure S27. Compared with the other groups, the ATP level of OFCCF + group decreased significantly owing to Ca^2+^ overload in mitochondria, which would damage the energy supply of cancer cells (Additional file 1: Figure S28). To identify the ability of OFCCF to relieve tumor hypoxia, mice tumors were obtained for HIF-1α (green) staining assay after being injected with the nanoparticles for 10 h. As illustrated in the Fig. 6g, the intensity of fluorescence was hardly visible in OFCCF + group. For ROS staining assay, OFCCF + group showed the strongest DCFH-DA fluorescence signals in tumor slices, which was consistent with the HIF-1α (green) staining assay results. Meanwhile, the remission of tumor hypoxia was further verified by down-regulation of HIF-1α expression level, indicating that released oxygen could relieve intratumoral hypoxia and enhance production of ROS (Additional file 1: Figure S29).

To assess the potential therapeutic toxicity of FCCF, a systemic toxicity study was performed. The physiological pathology of main organs were analysed by H&E staining. No significant pathological changes in major organs (heart, liver, kidney lung, and spleen) were observed after different treatments (Additional file 1: Figure S30), suggesting the excellent biosafety of the nanocomposite in vivo. Meanwhile, biochemical analysis further confirmed that OFCCF + had low toxicity (Additional file 1: Figure S31).

## Conclusion

In summary, we have successfully prepared an OFCCF nanocomposite with two-way activation of MPTP opening by ROS and Ca^2+^ overload for precise targeted therapy of breast cancer. OFCCF with tumor-targeting ability could effectively aggregate at the tumor sites. The subsequent rapid release of Ca^2+^ and oxygen were attributed to the hypoxia and low pH of TME. Overflowed Ca^2+^ rapidly accumulated inside the cytoplasm, which led to Ca^2+^ influx and activated MPTP opening. Upon NIR light irradiation, the released oxygen could enhance the effect of COF-mediated PDT, and the generation of ROS could promote the activation of MPTP opening. Notably, these two activation mechanisms ensured the sustained opening of MPTP, leading to the change of mitochondria transmembrane potential, release of Cyt c and activation of caspases 3, thus inducing cell apoptosis. Additionally, for the first time, we utilized FeCOF with hypoxia responsive as oxygen carrier, alleviating tumor hypoxia and enhance PDT efficiency. More importantly, OFCCF nanocomposites based treatment has been proved to be a safe and effective strategy to inhibit tumor growth and open new directions for clinical cancer therapy.

## Supplementary Information

**Additional file 1: Table S1. **Size of Nanoparticles.** Figure S1. **The SEM images of FeCOF. Figure S2. The SEM images of FeCOF@CaCO_3_.** Figure S3. **(a) XPS spectra of FeCOF@CaCO_3_. (b) XPS high-resolution scans of Ca 2p.** Figure S4.** The PXRD of FeCOF and FeCOF@CaCO_3_.** Figure S5. **(a) N_2_ adsorption and desorption isotherms at 77 K of FeCOF and FeCOF@CaCO_3_. (b)Pore size distribution plots of FeCOF and FeCOF@CaCO_3_.** Figure S6.** The Hydrodynamic diameters of FeCOF, FeCOF@CaCO_3_ and FCCF.** Figure S7.** The zate potential of FeCOF, FeCOF@CaCO_3_ and FCCF.** Figure S8.** (a) The FCCF dispersed in water, PBS and cell culture medium (containing 10% serum) before and after 7 days incubation. (b) The TEM image of FCCF stored in cell culture medium (containing 10% serum) for a week. (c) The DLS was measured on day 0, 5 and 7 when FCCF was dispersed in water, PBS and cell culture medium (containing 10% serum).** Figure S9.** (a) SEM images of FCCF at pH 7.4, 6.5 and 5.5, related to Figure 2a. Data are presented as mean ± SD (n = 3).** Figure S10.** (a) SEM images of FeCOF at pH 7.4, 6.5 and 5.5. Data are presented as mean ± SD (n = 3). Figure S11. Oxygen release behaviour of OFCCF in solution.** Figure S12. **CLSM microimages of intracellular Ca^2+^ content in 4T1 cells. Figure S13. CLSM microimages of mitochondrial Ca^2+^ content in 4T1 cells.** Figure S15.** Quantitative analysis of the intracellular O_2_ generation of 4T1 cells after various treatments based on the confocal images shown in Figure 3b by using imageJ, related to Figure 3b.** Figure S16.** Quantitative analysis of HIF-1*α* protein expression, as the ratio of protein to β-actin from Western Blot results. Related to Figure 3c. *P* values were calculated by one-way analysis (*p<0.05, **p<0.01, ***p<0.001).** Figure S17. **Intracellular ROS production of 4T1 cells under normoxic and hypoxic conditions after various treatments.** Figure S18.** MPTP opening in mitochondria of 4T1 cells after different treatments.** Figure S19.** mitochondrial membrane potential images of 4T1 cells after different treatments.** Figure S20.** Changes of intracellular ATP contents of 4T1 cells after being treated with PBS, L, COF+, FCCF, FCCF+ and OFCCF+ for 24 h. *P* values were calculated by one-way analysis (*p<0.05, **p<0.01, ***p<0.001). Data were represented as mean ± SD (n = 4).** Figure S21.** Relative viabilities of L929 cells treated by incubation with COF and FCCF at different concentration for (a) 24 h and (b) 48 h. Data were represented as mean ± SD (n = 6).** Figure S22.** The images of 4T1 cells after various treatments and stained with calcein-AM (live cells: green) and PI (dead cell: red).** Figure S23.** Quantitative analysis of Annexin V-FITC/PI co-stained 4T1 cells at 24 h after various treatments.** Figure S24. **Changes of body weight of mice in different groups after treatment.** Figure S25.** The tumor picture of mice before and after treatment in different groups (n = 8).** Figure S26.** (a) The photos of lung at the end of various treatments. (b) H&E-stained lung sections at the end of various treatments. Red arrows indicate the sites of tumor metastasis.** Figure S27. **Quantitative analysis of caspase 3, Bcl-2 and Cyt c protein expression, as the ratio of protein to β-actin from Western Blot results. Related to Figure 6 (f).** Figure S28.** ATP contents of tumor after being treated with PBS, L, COF+, FCCF, FCCF+ and OFCCF+. *P* values were calculated by one-way analysis (*p<0.05, **p<0.01, ***p<0.001). Data are presented as mean ± SD (n = 4).** Figure S29.** (a) The HIF-1*α *protein expression of tumor via western blotting analysis. (b) Quantitative analysis of HIF-1*α* protein expression, as the ratio of protein to β-actin from Western Blot results. *P *values were calculated by one-way analysis (*p<0.05, **p<0.01, ***p<0.001).** Figure S30. **H&E stained images of the major organs, including heart, liver, spleen, lung, and kidney. Scale bar: 20 μm.** Figure S31.** BALB/c mice were sacrificed after PBS and FCCF (NPs) treatment. Serum biochemistry data including Alanine aminotransferase (ALT), Aspartate aminotransferase (AST) and Alkaline phosphatase (ALP) as hepatic function indicators and Blood urea nitrogen (BUN) as renal function indicators were measured. Blood hematological counts: Blood levels of White blood cells (WBC), Red blood cells (RBC), Hemoglobin (HGB), Hematocrit (HCT), Mean corpuscular volume (MCV), Mean corpuscular hemoglobin (MCH), Mean corpuscular hemoglobin concentration (MCHC) and Blood platelet (PLT). Data are presented as mean ± SD (n = 4).

## Data Availability

The data that support the findings of this study are available from the corresponding author upon reasonable request. Some data may not be made available because of privacy or ethical restrictions**.**
