# Supplementary material for: Double-activation of mitochondrial permeability transition pore opening via calcium overload and reactive oxygen species for cancer therapy
Source: J Nanobiotechnology. 2022 Apr 12;20:188. doi: 10.1186/s12951-022-01392-y (PMC9004178; doi:10.1186/s12951-022-01392-y)
Supplement: Supplementary file 1 — Additional file 1: Table S1. Size of Nanoparticles. Figure S1. The SEM images of FeCOF. Figure S2. The SEM images of FeCOF@CaCO3. Figure S3. (a) XPS spectra of FeCOF@CaCO3. (b) XPS high-resolution scans of Ca 2p. Figure S4. The PXRD of FeCOF and FeCOF@CaCO3. Figure S5. (a) N2 adsorption and desorption isotherms at 77 K of FeCOF and FeCOF@CaCO3. (b)Pore size distribution plots of FeCOF and FeCOF@CaCO3. Figure S6. The Hydrodynamic diameters of FeCOF, FeCOF@CaCO3 and FCCF. Figure S7. The zate potential of FeCOF, FeCOF@CaCO3 and FCCF. Figure S8. (a) The FCCF dispersed in water, PBS and cell culture medium (containing 10% serum) before and after 7 days incubation. (b) The TEM image of FCCF stored in cell culture medium (containing 10% serum) for a week. (c) The DLS was measured on day 0, 5 and 7 when FCCF was dispersed in water, PBS and cell culture medium (containing 10% serum). Figure S9. (a) SEM images of FCCF at pH 7.4, 6.5 and 5.5, related to Figure 2a. Data are presented as mean ± SD (n = 3). Figure S10. (a) SEM images of FeCOF at pH 7.4, 6.5 and 5.5. Data are presented as mean ± SD (n = 3). Figure S11. Oxygen release behaviour of OFCCF in solution. Figure S12. CLSM microimages of intracellular Ca2+ content in 4T1 cells. Figure S13. CLSM microimages of mitochondrial Ca2+ content in 4T1 cells. Figure S15. Quantitative analysis of the intracellular O2 generation of 4T1 cells after various treatments based on the confocal images shown in Figure 3b by using imageJ, related to Figure 3b. Figure S16. Quantitative analysis of HIF-1α protein expression, as the ratio of protein to β-actin from Western Blot results. Related to Figure 3c. P values were calculated by one-way analysis (*p<0.05, **p<0.01, ***p<0.001). Figure S17. Intracellular ROS production of 4T1 cells under normoxic and hypoxic conditions after various treatments. Figure S18. MPTP opening in mitochondria of 4T1 cells after different treatments. Figure S19. mitochondrial membrane potential images of 4T1 cells [file 12951_2022_1392_MOESM1_ESM.doc]

Additional file 1

Double-activation of mitochondrial permeability transition pore opening via calcium overload and reactive oxygen species for cancer therapy

Ying Zhou^1,3†^, Shisong Jing^2†^, Sainan Liu^3,4^, Xizhong Shen^5^, Lihan Cai^3,4^, Changfeng Zhu^5*^, Yicheng Zhao^1,6*^ and Maolin Pang^3,4*^

^1^Center for Pathogen Biology and Infectious Diseases, Key Laboratory of Organ Regeneration and Transplantation of the Ministry of Education, The First Hospital of Jilin University, Changchun 130021, China.

^2^College of Animal Science, School of Pharmacy, Jilin University, Changchun 130022, China.

^3^ State Key Laboratory of Rare Earth Resource Utilization, Changchun Institute of Applied Chemistry, Chinese Academy of Science, Changchun 130022, China.

^4^ University of Science and Technology of China, Hefei 230026, China.
^5^Department of Gastroenterology and Hepatology, Zhongshan Hospital, Fudan University, Shanghai 200032, China; Shanghai Institute of Liver Diseases, Shanghai 200001, China.

^6^Clinical Medical College, Changchun University of Chinese Medicine, Changchun, Jilin 130117, China.

**Experimental Section**

Materials and Reagents

2,5-dimethoxyterephthaldehyde (C_10_H_10_O_4_, AR, Jilin Chinese Academy of Sciences-Yanshen Technology Co. Ltd), 1,3,5-tris(4-aminophenyl)benzene (C_24_H_21_N_3_, AR, 98%, Alpha), Ethanol (AR, Beijing Chemical Works), Acetonitrile (CH_3_CN, 99.8%, Vetec), Acetic acid (CH_3_COOH, AR, Beijng Chemical Works), Folic acid (C_19_H_19_N_7_O_6_, 98%, Solarbio), Iron(II) chloride tetrahydrate (FeCl_2_^.^4H_2_O, AR, 99.0%, Aladdin), Calcium chloride (CaCl_2_, 96%, Vetec), N-hydroxysuccinimide (NHS) and 1-ethyl-3-[3-(dimethylamino) propyl] carbodiimide hydrochloride (EDC) were obtained from J&K Scientific Ltd. 1,3-diphenylisobenzofuran (DPBF) and [Ru(dpp)_3_]Cl_2_ (RDPP) were purchased from Aladdin.

3-(4,5-Dimethylthiazolyl-2)-2,5-diphenyltetrazolium bromide (MTT), Rhod-2 AM, and 2′,7′-dichlorodihydrofluorescein diacetate (DCFH-DA) were purchased from Dalian Meilun Biotechnology Co., Ltd. (Dalian, P. R. China). Mitochondrial membrane potential assay kit with mitochondrial probe 5,5′,6,6′-tetrachloro-1,1′,3,3′-tetraethylbenzimidazolyl-carbocyanine iodide (JC-1), Fluo-4 AM, Mito Tracker® Red CMXRos, Mitochondrial Permeability Pore Assay Kit, ATP Assay Kit and Hoechst 33342 were purchased from Beyotime (Shanghai, P. R. China). All antibodies were supplied by Biolegend (Beijing, P. R. China).

Characterization

Powder X-ray diffraction (PXRD) studies were performed on a Rigaku MiniFlex 600 diffractometer with graphite monochromatized Cu Kα radiation (λ = 0.15405 nm). The sample was scanned at a scanning rate of 8°/min in the 2θ range from 2 to 20° at room temperature. Field emission scanning electron microscope (FE-SEM, S-4800, Hitachi) equipped spectrometer was used to characterize the morphology of the sample. Transmission electron microscopy (TEM) images were obtained on a FEI Tecnai G2 S-Twin with a field emission gun operating at 200 kV. Thermogravimetric analysis data was recorded on a TGA 500 thermogravimetric analyzer by heating with a rate of 10°C/min under the nitrogen atmosphere (60 mL min^-1^). Fourier transform infrared spectroscopy (FT-IR) was measured on a Vertex PerkinElmer 580 BIR spectrophotometer (Bruker) using the KBr tabletting technique. The sample was securely packaged to obtain a transparent film. The UV-Vis adsorption spectral values were obtained on a U-3310 spectrophotometer (Hitachi). The X-ray photoelectron spectra (XPS) were taken on a VG ESCALAB MK II electron energy spectrometer using Mg KR (1253.6 eV) as the X-ray excitation source. Dynamic light scattering (DLS) experiment was measured by Malvern Zeta Sizer-Nano ZS90 instrument at 25°C. MTT experiments were carried out using a microplate reader (Thermo Multiskan MK3). Part of fluorescent images and videos were taken by confocal laser scanning microscope (ZEISS LSM 980).

Statistical Analysis

The data are shown as mean ± SD. The significance of the difference was determined by one-way analysis of variance (**p*<0.05, ***p*<0.01, ****p*<0.001).

Materials Synthesis

Synthesis of FeCOF: 0.25 mL FeCl_2_ solution (50 mg mL^-1^), 10 mg of DMTP and 10 mg of TAPB were dissolved in 20 mL of acetonitrile separately, and then the two solutions were mixed. Subsequently, 0.5 mL of acetic acid was added and the mixture was stirred at room temperature for 12 h. The precipitate was collected by centrifugation and washed with ethanol for three times.

Synthesis of FeCOF@CaCO_3_: FeCOF@CaCO_3_ nanoparticles were synthesized by a gas diffusion reaction. Briefly, 44 mg of CaCl_2_ was added to 10 mg of FeCOF ethanol and deionized water mixed solutions (V/V=1:1). Then, the bottle was put into a vacuum drying chamber containing 5 g dry ammonia bicarbonate (NH_4_HCO_3_) at 40^o^C. After keeping the whole system in a vacuum environment for 8 h, FeCOF@CaCO_3_ nanoparticles were obtained and could be separated by centrifugation at 8000 rpm. Those nanoparticles were re-dispersed in deionized water for further modification.

Synthesis of FeCOF@CaCO_3_@FA: FeCOF@CaCO_3_ (5 mg), EDC (10 mg) and NHS (10 mg) were dissolved in 5 mL of deionized water. After reaction for 1 h, 5 mL of FA solution (1 mg mL^-1^) was mixed with the above solution and stirred for 24 h at room temperature.

Synthesis of O_2_-FeCOF@CaCO_3_@FA: 5 mg FeCOF@CaCO_3_@FA was dispersed in 10 mL deionized water. After pumping with O_2_ for 1 h, the products were directly used in subsequent expriments.

Extracellular O_2_ detection: For the O_2_ production ability of OFCCF, the OFCCF (5 mg) was added into deionized water. Then, the concentration of generated O_2_ was measured by a portable dissolved oxygen meter.

PH-triggered release of Ca^2+^ from FCCF: The FCCF (1mg) was dissolved in 5 mL PBS solution (pH = 5.5, 6.5 and 7.4), and was put in a shaking incubator (37°C, 150 rpm) for a determined period. Then, the supernatant solution was collected by centrifugation for measuring the amount of released Ca^2+^ by inductively coupled plasma-optical emission spectroscopy (ICP-OES). The morphology changes of FCCF at different pH were observed via SEM. The DLS was measured on day 0, 5 and 7 when FCCF was dispersed in water, PBS and cell culture medium (containing 10% serum).

Extracellular ROS detection: For the ROS production ability of OFCCF, the 1,3-diphenylisobenzofuran (DPBF) (5 mg mL^-1^ in DMSO, 10 μL) was chose as an indicator to detect the production of ^1^O_2_. The OFCCF (100 μg mL^-1^) was dispersed in 2 mL of deionized water and 10 µL of DPBF solution was added. Then the mixed solution was irradiated with a 650 nm laser for different times (0, 3, 6, 9, 12, 15 minutes). The decrease of DPBF absorption around 410 nm was monitored by UV-Vis spectroscopy at different time intervals. Besides, the 2,2,6,6-Tetramethylpiperidine (TEMP) was used as a capture agent to detect the generation of ^1^O_2_ by electron spin resonance (ESR) spectrometer.

Cell lines and animals: Mouse fibroblast cell line (L929) and murine breast tumor cell line (4T1) were chosen for cell tests. L929 and 4T1 were purchased from the Institute of Biochemistry and Cell Biology, Chinese Academy of Sciences. L929 cells were first cultured in Dulbecco’s Modified Eagle’s Medium (DMEM) supplemented with 10% heat-inactivated fetal bovine serum (FBS, GIBCO), 100 units per mL of penicillin and 100 units per mL of streptomycin (Sigma) in an atmosphere of 5% CO_2_ at 37°C. 4T1 cells were first cultured in Roswell Park Memorial Institute (RPMI/1640) supplemented with 10% heat-inactivated FBS, 100 units per mL of penicillin and 100 units per mL of streptomycin in an atmosphere of 5% CO_2_ at 37°C. (If there is no special instruction, cell experiments are performed under hypoxic conditions).

Female BALB/c mice (18-20 g) were purchased from the Center for Experimental Animals, Jilin University (Changchun, China). All mice were handled using the protocol approved by the Institutional Animal Care and Use Committee of Jilin University.

Endo/lysosome escape: 4T1 cells were seeded in 20 mm wells of a glass bottom cell culture dish (10^5^ cells per well) and cultured overnight. Rhodamine B (RhB)-labeled FCCF (100 μg mL^-1^) was added to per well and incubated for 1, 3, 6 h. According to the manufacturer's instructions, the lysosomes were labelled with LysoTracker@Green (Invitgen). Next, 4T1 cells were stained with Hoechst33342 for 10 min and observed by confocal laser scanning microscope (CLSM).

MTT assay: In order to evaluate the cytocompatibility of OFCCF and COF, L929 and 4T1 cells were incubated with different concentrations of OFCCF and COF (0, 1.56, 3.125, 6.25, 12.5, 25, 50, 100, 200, 400 μg mL^-1^) for 24 h and 48 h, respectively. The relative cell viability was calculated by methyl thiazolyl tetrazolium (MTT) assay.

The 4T1cells were planted into a 96-well plate and cultured under hypoxia and normoxia, respectively. Then the OFCCF, FCCF and COF (100 μg mL^-1^) were added to the culture medium. After 4 h of incubation, the 10 μL of 3-(4, 5-dimethylthiazol-2-yl)-2, 5- diphenyltetrazolium bromide (MTT) was added into every well. After 4 h, 150 μL of dimethyl sulfoxide (DMSO) was added into every well. The relative cell viability was calculated by

Ca^2+^ contents detection: To visualize the intracellular Ca^2+^ of 4T1 cells via CLSM, The cells were seeded in 20 mm wells of a glass bottom cell culture dish (10^5^ cells per well) in 1 mL of FPS-1640 and cultured for 24 h. Then, the medium was replaced with (a) PBS, (b) L, (c) FCCF (100 μg mL^-1^), (d) COF+ (100 μg mL^-1^), (e) FCCF+ (100 μg mL^-1^), (f) OFCCF+ (100 μg mL^-1^), (g) CaCO_3_ (Ca^2+^, 10 μg) for 6 h. After washing with PBS, 4T1 cells were stained with Fluo-4 AM and detected by CLSM.

For visualizing mitochondrial Ca^2+^ inside 4T1 cells via CLSM, the cells were seeded in 20 mm wells of a glass bottom cell culture dish (10^5^ cells per well) in 1 mL of FPS-1640 and cultured for 24 h. Then, the medium was replaced with 2.0 mL of FPS-1640 with (a) PBS, (b) L, (c) FCCF (100 μg mL^-1^), (d) COF+ (100 μg mL^-1^), (e) FCCF+ (100 μg mL^-1^), (f) OFCCF+ (100 μg mL^-1^), (g) CaCO_3_ (Ca^2+^, 10 μg) for 6 h. After washing with PBS, the cells were stained with Rhod-2 AM and detected by CLSM.

To detect the intracellular Ca^2+^ concentration, the Calcium Colorimetric assay kit was purchased from Beyotime Biotechnology, and the OD_575_ value was measured.

Intracellular reactive oxygen species (ROS) detection: 2′,7′-dichlorofluorescin diacetate (DCFH-DA) was used as a probe to detect ROS production *in vitro*. Firstly, 4T1 cells were seeded into in 20 mm wells of a glass bottom cell culture dish (10^5^ cells per well) overnight and treated with (a) PBS, (b) L, (c) FCCF (100 μg mL^-1^), (d) COF+ (100 μg mL^-1^), (e) FCCF+ (100 μg mL^-1^), (f) OFCCF+ (100 μg mL^-1^) under the normoxia and hypxia. After 6 h of incubation in the dark, 2′,7′-dichlorofluorescin diacetate was added to each well and the mixture was incubated for 30 min at 37^o^C. Then, the cells were imaged by CLSM.

Intracellular O_2_ detection: The intracellular O_2_ release ability of OFCCF was investigated with [Ru(dpp)_3_]Cl_2_ (RDPP) probe under the hypxia. 4T1 cells were seeded in 20 mm wells of a glass bottom cell culture dish (10^5^ cells per well) and incubated for 24 h. After 6 h of treatment with (a) PBS+, (b) FCCF (100 μg mL^-1^), (c) FCCF+ (100 μg mL^-1^), (d) OFCCF+ (100 μg mL^-1^). Then the cells were incubated with RDPP (0.1 μM) for 4 h. The stained cells were washed 3 times with PBS, and the cells were imaged by CLSM.

Mitochondrial permeability transition pore (MPTP) assay: The opening degree of mitochondrial permeability transition pore was detected with Calcein AM or Calcein AM + CoCl_2_ probe. 4T1 cells were cultured in a culture medium containing COF (100 μg mL^–1^), FCCF (100 μg mL^–1^) and OFCCF (100 μg mL^–1^) for 6 h and treated with different groups: (a) PBS+, (b) FCCF, (c) COF+, (d) FCCF+, (e) OFCCF+, (f) CCCP. The cells were incubated with Calcein AM and CoCl_2_ for 1 h and imaged by CLSM.

Mitochondrial membrane potential (JC-1) detection: For *in vitro* mitochondrial membrane potential change detection, 4T1 cells were then treated with different groups: (a) PBS, (b) L, (c) FCCF (100 μg mL^-1^), (d) COF+ (100 μg mL^-1^), (e) FCCF+ (100 μg mL^-1^), (f) OFCCF+ (100 μg mL^-1^), (g) CaCO_3_ (Ca^2+^, 10 μg). After being incubated for 4 h, the culture medium was replaced with JC-1 staining solution according to the manufacturer’s protocol. The cells were then washed three times with PBS. Finally, the cells were imaged by CLSM.

Observation of mitochondrial distribution: For measuring mitochondria distribution, 4T1 cells were seeded in 20 mm wells of a glass bottom cell culture dish (10^5^ cells per well) in 1 mL of FPS-1640 and cultured for 24 h. Then, the medium was replaced with (a) PBS, (b) L, (c) FCCF (100 μg mL^-1^), (d) COF+ (100 μg mL^-1^), (e) FCCF+ (100 μg mL^-1^), (f) OFCCF+ (100 μg mL^-1^), (g) CaCO_3_ (Ca^2+^, 10 μg) for 6 h. After washing with PBS, the cells were stained with Mito Tracker® Red CMXRos and detected by CLSM.

Observation of mitochondrial morphology: To measure mitochondrial morphology changes, 4T1 cells were seeded in 100 mm × 20 mm dish at a density of 3.0 × 10^6^ cells per dish in 4.0 mL of FPS-1640 and cultured for 24 h. Then, the medium was replaced with 4.0 mL of FPS-1640 containing FCCF (100 μg mL^-1^), COF (100 μg mL^-1^), OFCCF (100 μg mL^-1^) and cultured for 12 h. After washing with PBS twice, cells were treated with trypsin and then centrifuged by 1,000 rpm to collect the precipitation. The obtained precipitation was fixed, dehydrated, embedded and sliced, and measured by biological transmission electron microscopy (Bio-TEM).

Adenosine triphosphate (ATP) detection: The ATP level *in vitro* was detected via ATP assay kit. 4T1 cells were seeded in 20 mm wells of a glass bottom cell culture dish (10^5^ cells per well) and cultured overnight under hypoxia and treated with different groups: (a) PBS, (b) L, (c) FCCF (100 μg mL^-1^), (d) COF+ (100 μg mL^-1^), (e) FCCF+ (100 μg mL^-1^), (f) OFCCF+ (100 μg mL^-1^). Then the cells were washed three times with PBS. In the end, the cells were lysed, centrifuged to take the supernatant and tested according to the instruction.

Live-dead cell staining experiments: 4T1 cells were seeded in 12-well plate at a density of 1 × 10^5^ cells per well and incubated for 24 h. Afterwards, the 4T1 cells were treated with (a) PBS, (b) L, (c) FCCF (100 μg mL^-1^), (d) COF+ (100 μg mL^-1^), (e) FCCF+ (100 μg mL^-1^), (f) OFCCF+ (100 μg mL^-1^). Then, Calcein-AM and pyridine iodide (PI) staining reagents were applied to stain the viable cells as green fluorescence (λex = 490 nm, λem = 515 nm) and dead cells as red fluorescence (λex = 535 nm, λem = 617 nm). The fluorescence was routinely detected using an inverted florescence microscope system (Nikon Ti–S).

Western blot: For western blots of HIF-1α, Bcl-2, Caspase 3 and Cyt c expressions, 4T1 cells were seeded into culture plates at a density of 3 × 10^6^ cells per dish and treated with (a) PBS+, (b) FCCF (100 μg mL^-1^), (c) FCCF+ (100 μg mL^-1^) and (d) OFCCF+ (100 μg mL^-1^) for 12 h, respectively. Subsequently, the cells were washed repeatedly with PBS and collected for a standard western blot process.

Mouse experiment model: The 4T1 cancer cells were subcutaneously injected into the back of BALB/c mice. When tumor volumes grew to about 60 mm^3^, the mice were randomly divided into 6 groups (n = 8 per group): (a) PBS, (b) L, (c) FCCF, (d) COF+, (e) FCCF+, (f) OFCCF+. Mice were injected intravenously with nanocomposites (100 µL, 1 mg mL^-1^) for 12 h and received 650 nm (0.72 W cm^-2^, 5 min) laser irradiation. The tumor sizes and weight were measured for every other day. The relative tumor volume was V/V_0_ and the tumor volume was calculated by V = 4/3 × length × width^2^/8, where V_0_ was the tumor volume before treatment.

*In vivo* Bio-distribution study: 4T1 tumor-bearing mice were intravenously injected with FCCF (100 µL, 1 mg mL^-1^).After different times (0 h, 1 h, 4 h, 12 h, 1 day, 3 days, and 7 days), main organs were collected, weighed and dissolved in digesting aqua regia. The contents of Fe in various samples were detected by ICP-MS. To investigate the amount of Ca^2+^ accumulated in various organs and tumor under 650 nm laser irradiation (0.72W cm^2^) after 12 h intravenous injection of PBS+, FCCF (100 µL, 1 mg mL^-1^), FCCF+ (100 µL, 1 mg mL^-1^) and OFCCF+ (100 µL, 1 mg mL^-1^). The contents of Ca^2+^ in various samples were detected by ICP-MS

Systemic toxicity in vivo: To evaluate the systemic toxicity, these mice were executed after therapy. The major organs (heart, liver, spleen, lung and kidney) and tumors were collected for H&E assay. Furthermore, the tumors were also assessed by TUNEL, caspase-3, HIF-1α and ROS staining. Meanwhile, the blood of these mice was gathered for biochemistry assay.

Western blot and ELISA in vivo: 4T1 tumor-bearing mice were treated with (a) PBS+, (b) FCCF (100 μg mL^-1^), (c) FCCF+ (100 μg mL^-1^), (d) OFCCF+ (100 μg mL^-1^). Tumor and tumor supernatant were collected and applied for the western blot (HIF-1α, Bcl-2, Caspase 3 and Cyt c) and ELISA (Apaf-1) measurement, respectively.


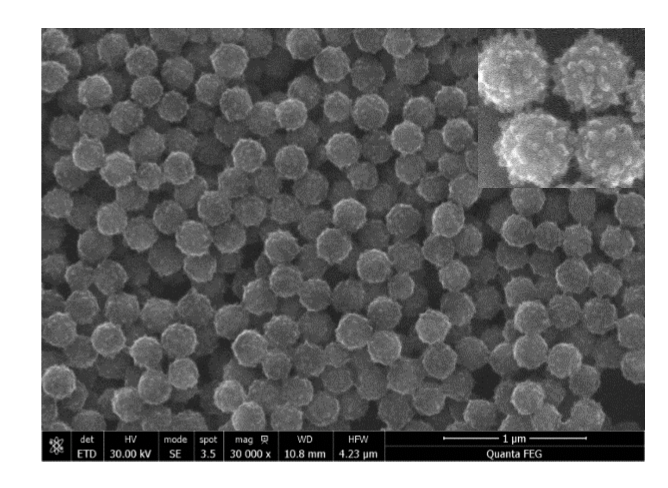
Table S1. Size of Nanoparticles


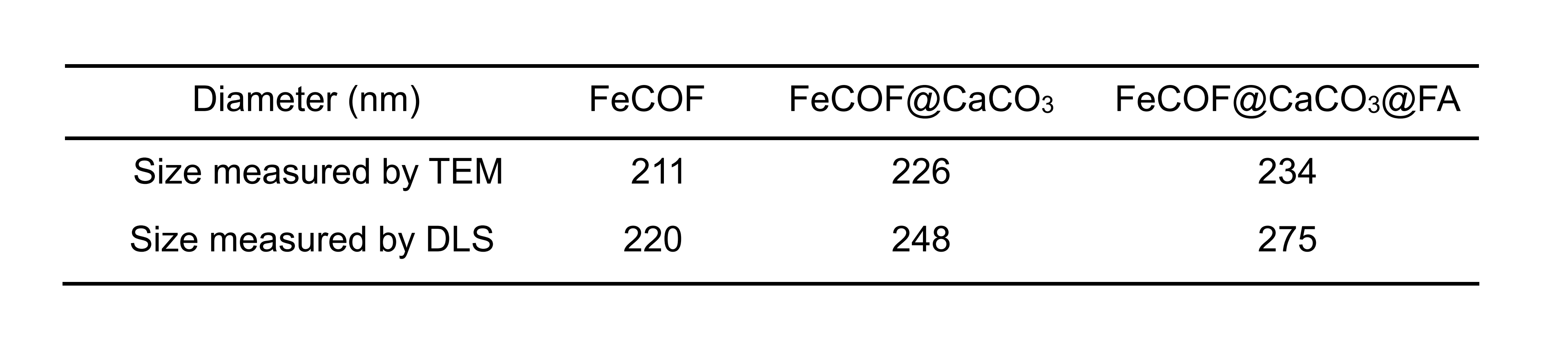
**Figure S1.** The SEM images of FeCOF.


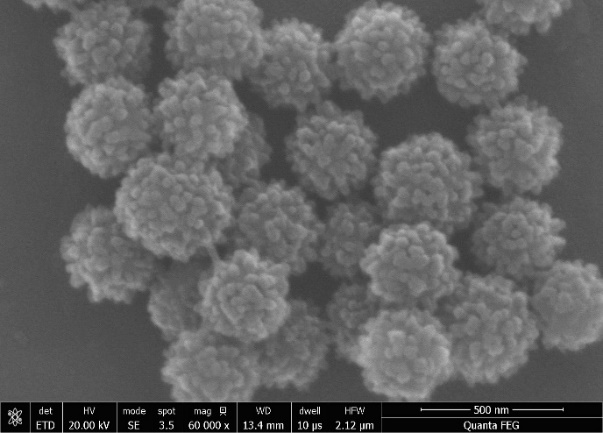
Figure S2. The SEM images of FeCOF@CaCO_3_.


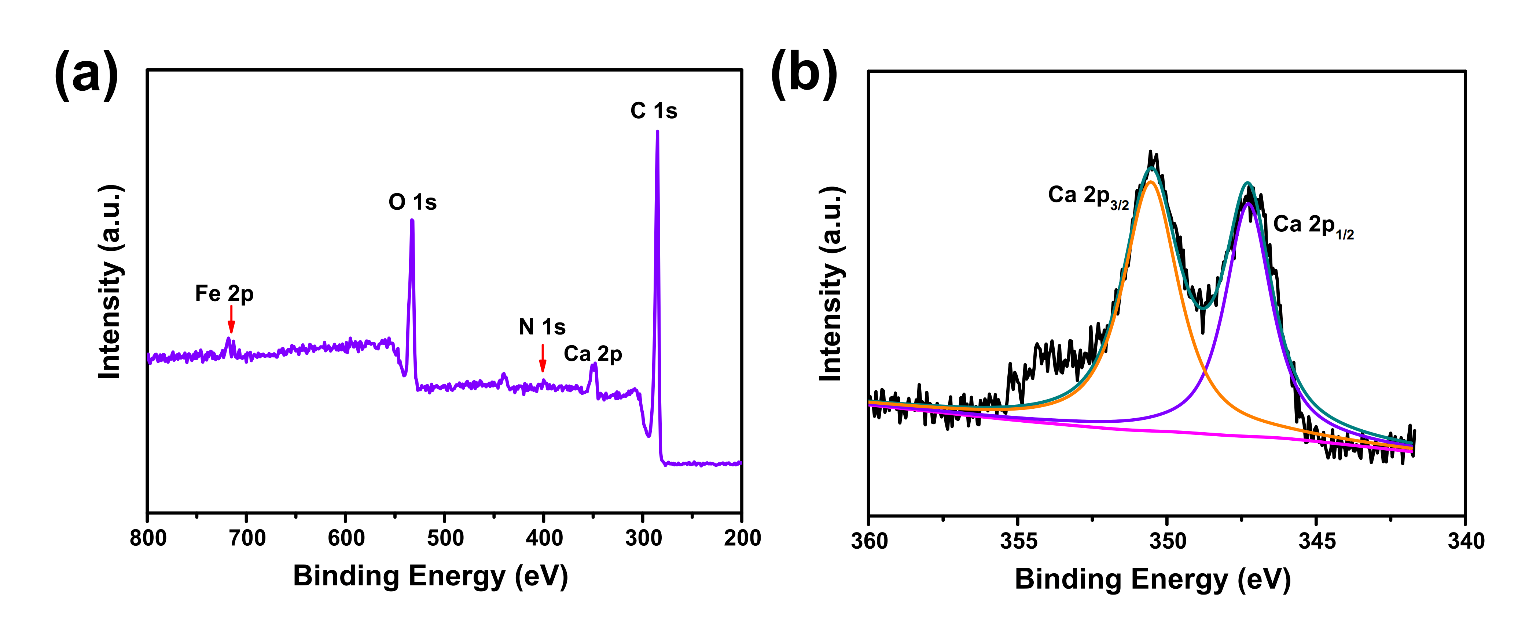
Figure S3. (a) XPS spectra of FeCOF@CaCO_3_. (b) XPS high-resolution scans of Ca 2p.


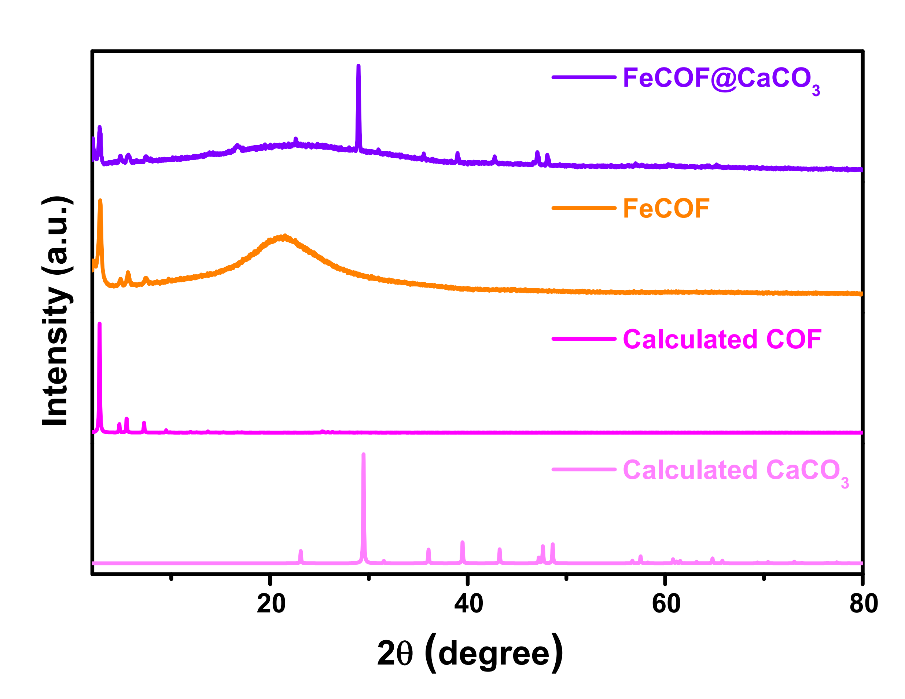
Figure S4. The PXRD of FeCOF and FeCOF@CaCO_3_.


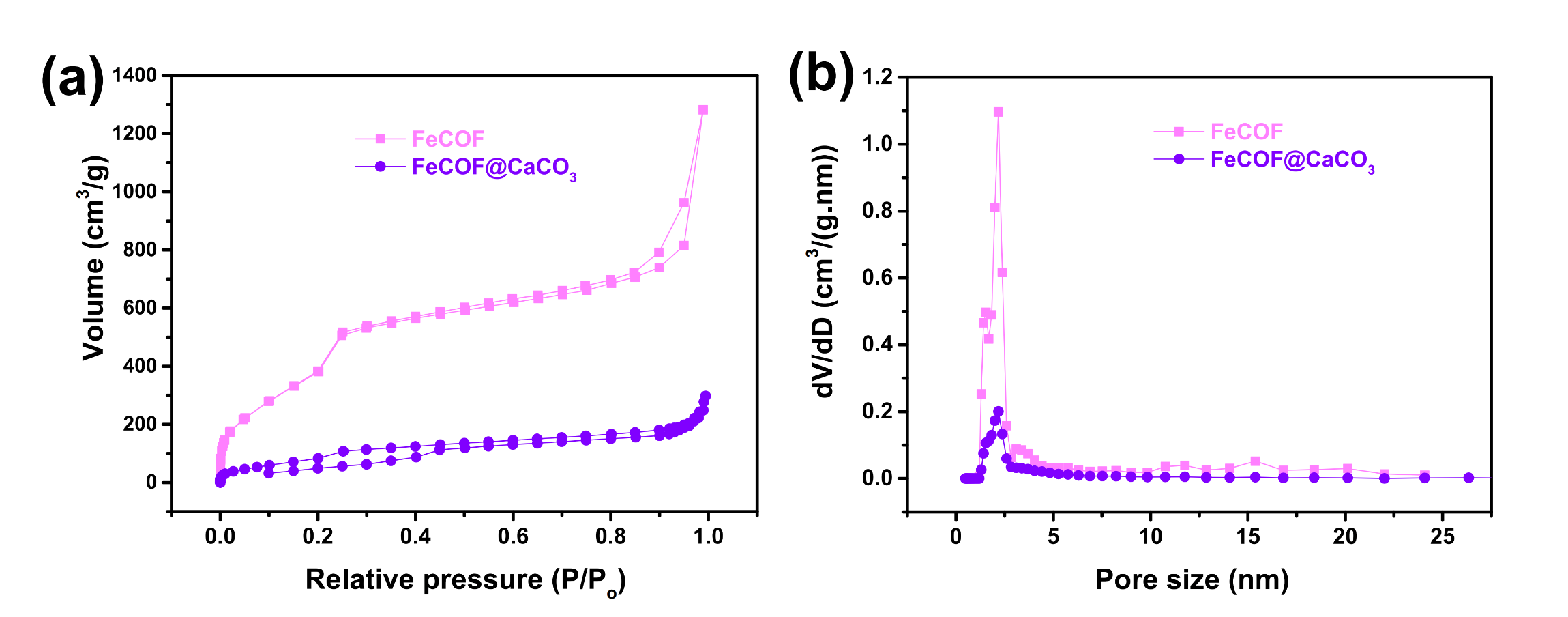
Figure S5. (a) N_2_ adsorption and desorption isotherms at 77 K of FeCOF and FeCOF@CaCO_3_. (b)Pore size distribution plots of FeCOF and FeCOF@CaCO_3_.


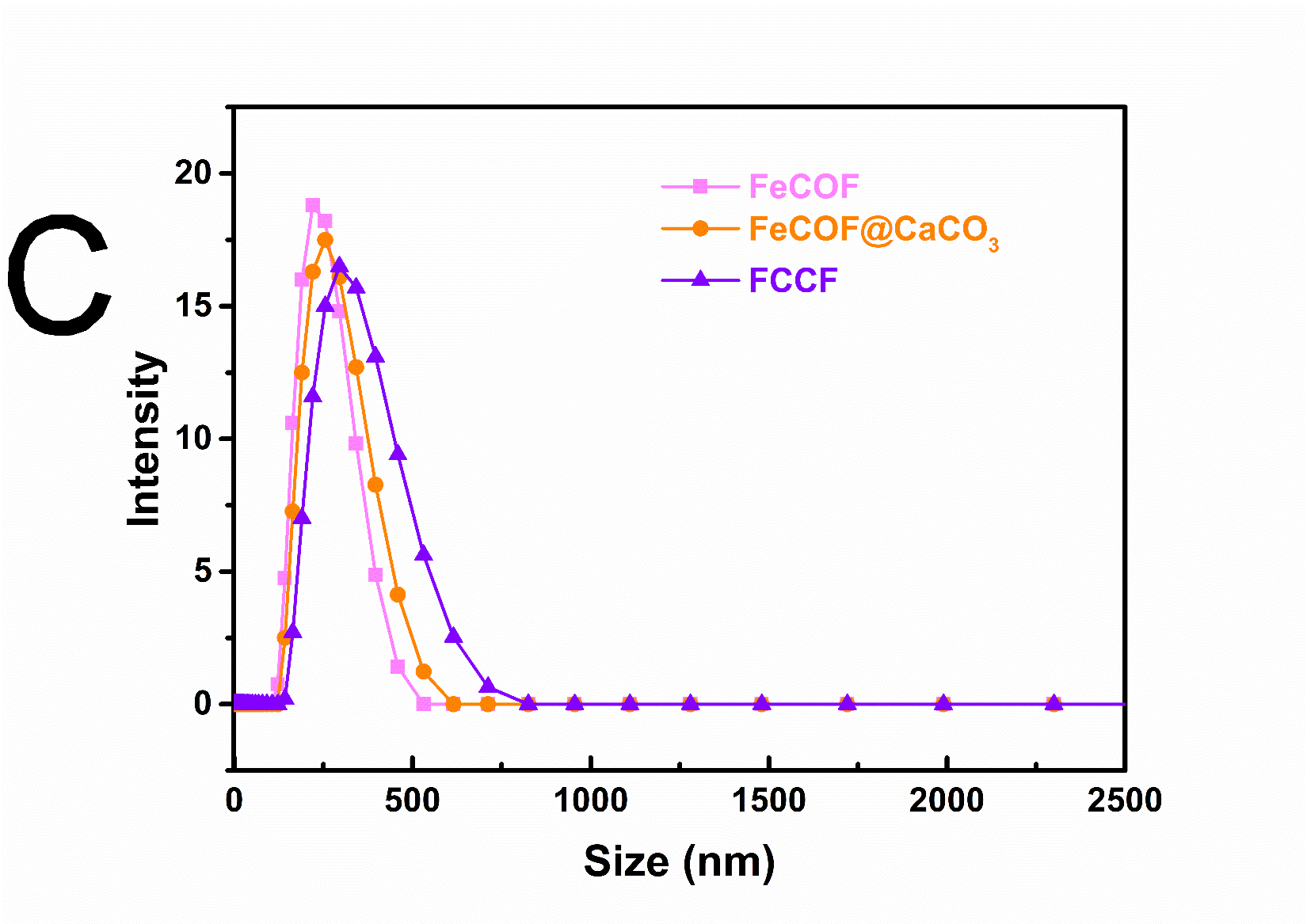


**Figure S6** The Hydrodynamic diameters of FeCOF, FeCOF@CaCO_3_ and FCCF.

#
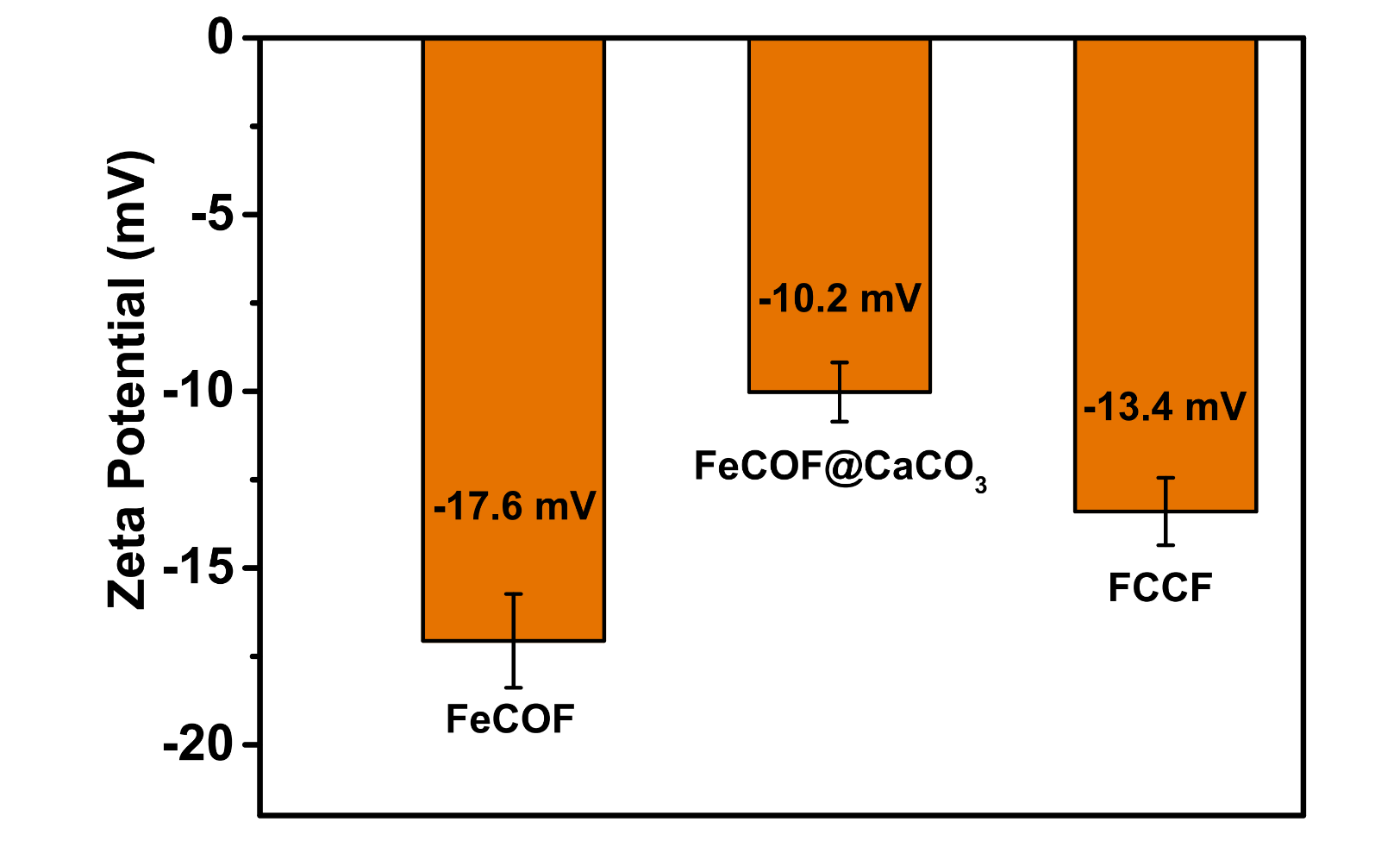
Figure S7. The zate potential of FeCOF, FeCOF@CaCO_3_ and FCCF.

**
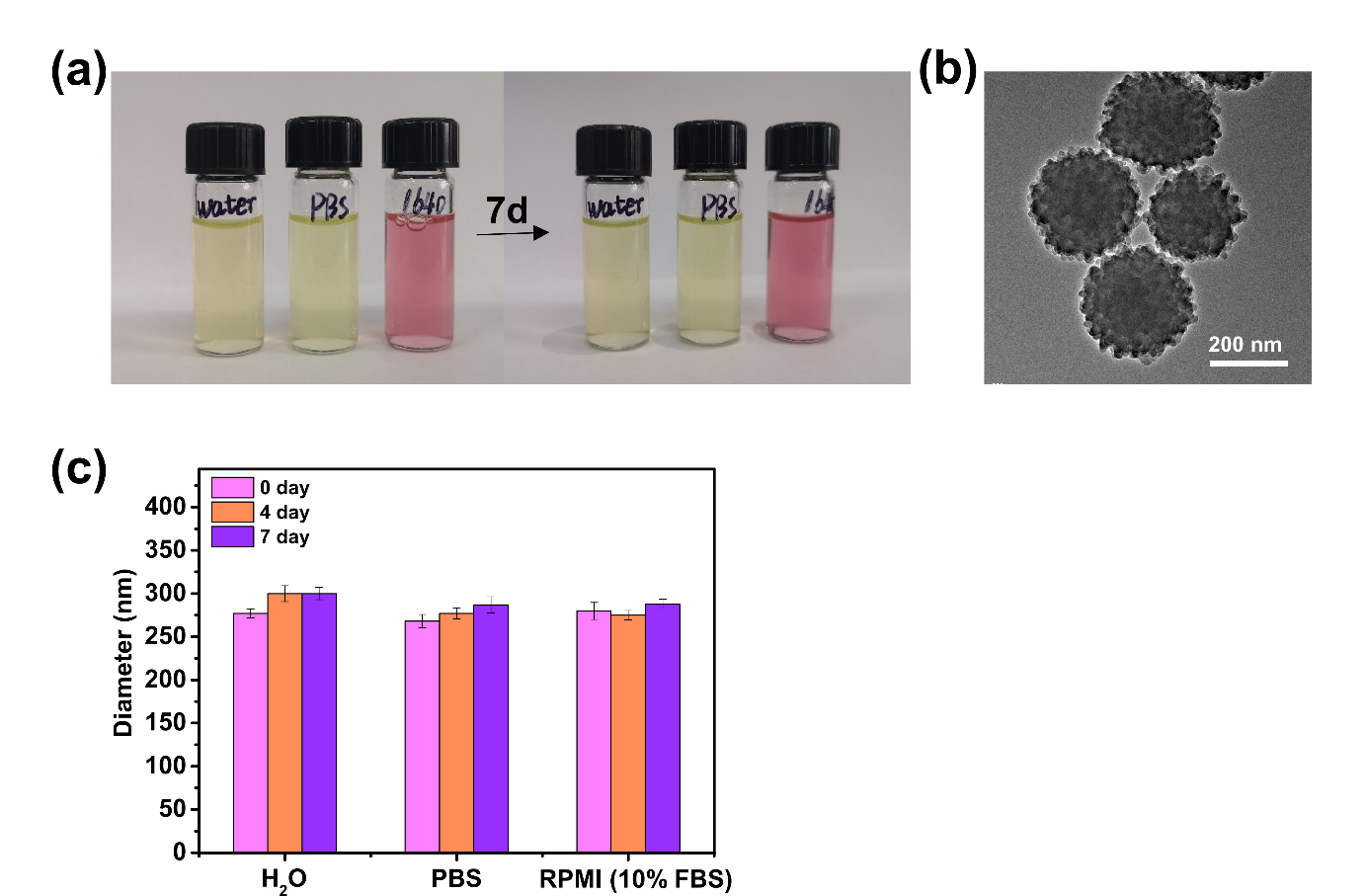
**

**Figure S8.** (a) The FCCF dispersed in water, PBS and cell culture medium (containing 10% serum) before and after 7 days incubation. (b) The TEM image of FCCF stored in cell culture medium (containing 10% serum) for a week. (c) The DLS was measured on day 0, 5 and 7 when FCCF was dispersed in water, PBS and cell culture medium (containing 10% serum).


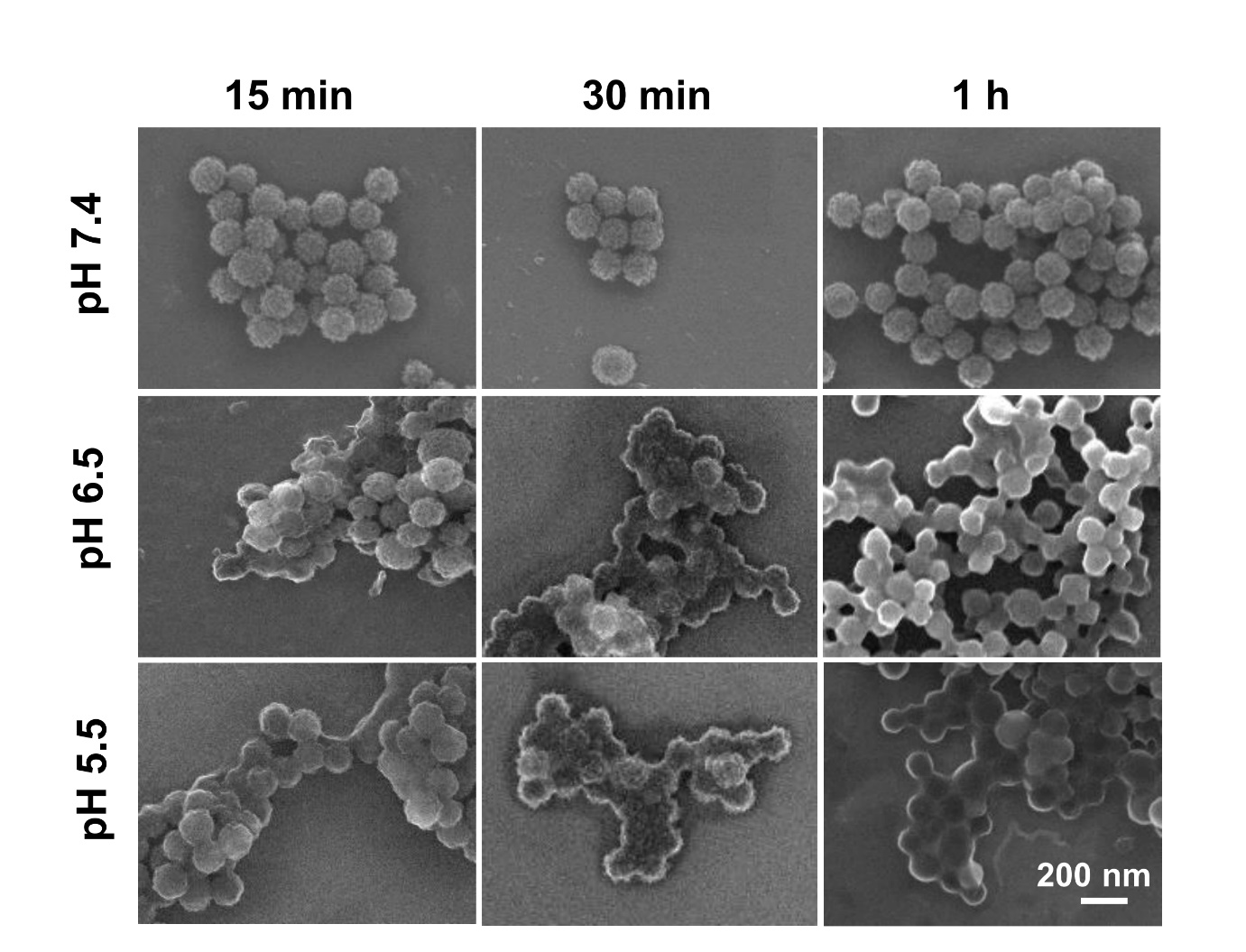


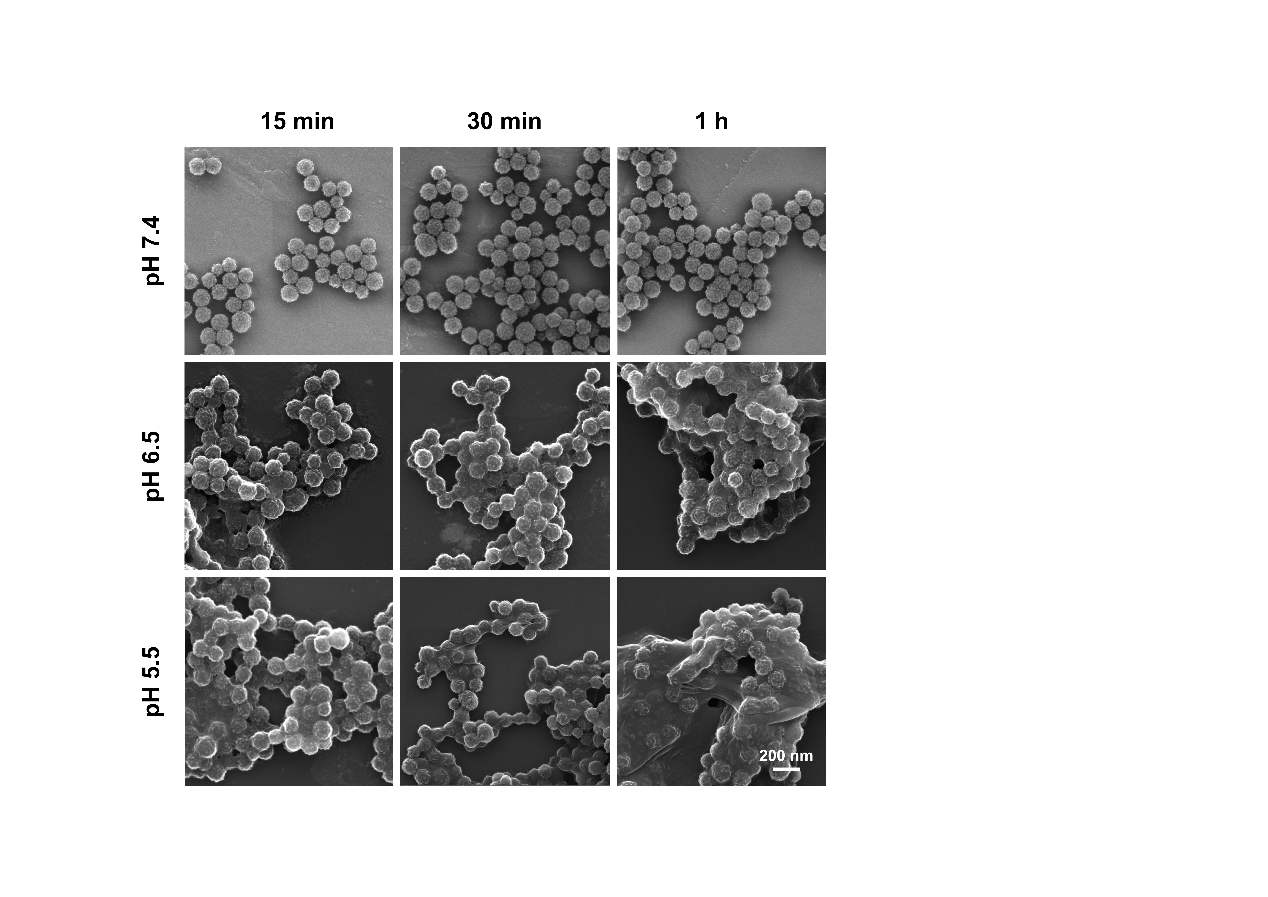
**Figure S9.** (a) SEM images of FCCF at pH 7.4, 6.5 and 5.5, related to Figure 2a. Data are presented as mean ± SD (n = 3).


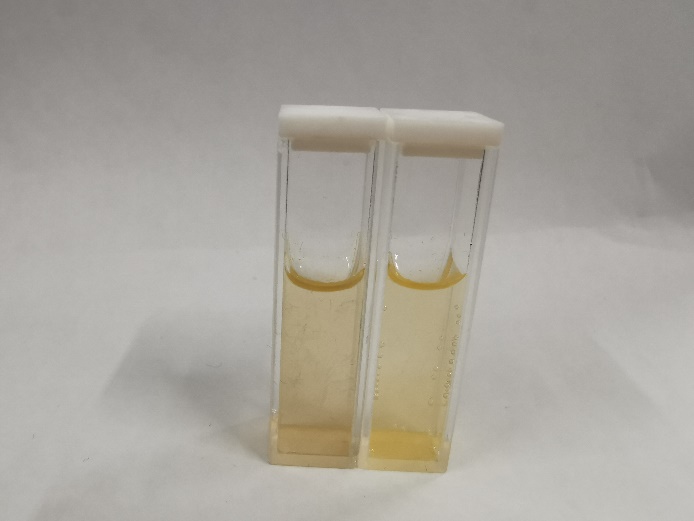
**Figure S10.** (a) SEM images of FeCOF at pH 7.4, 6.5 and 5.5. Data are presented as mean ± SD (n = 3).

**Figure S11**. Oxygen release behaviour of OFCCF in solution.


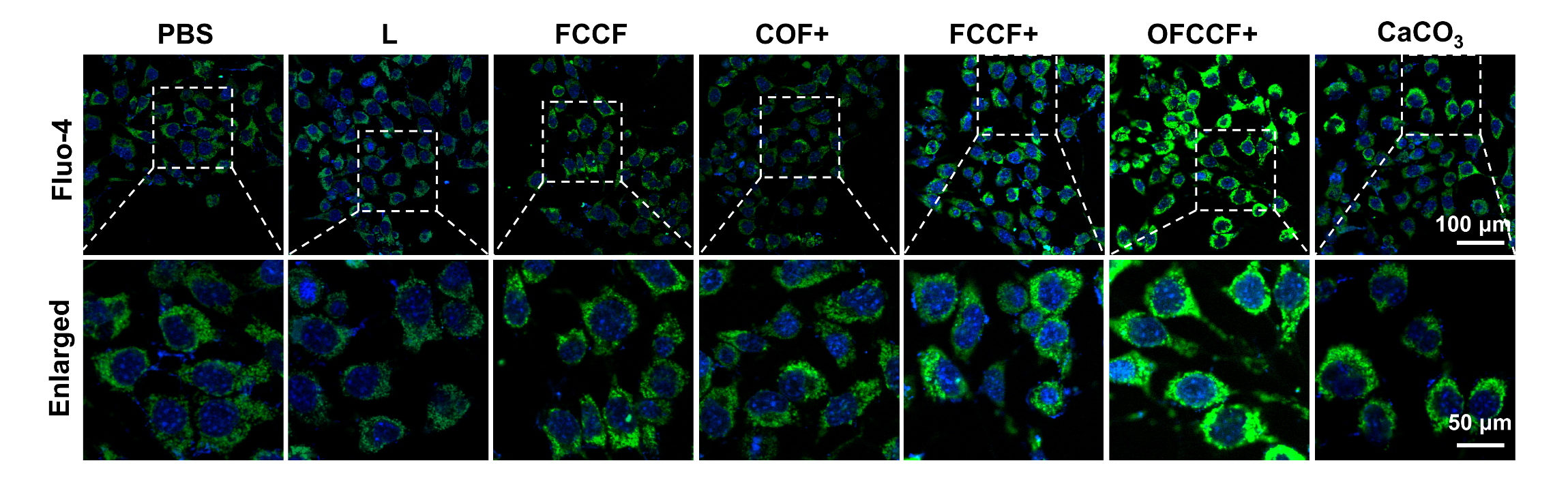
**Figure S12**. CLSM microimages of intracellular Ca^2+^ content in 4T1 cells.


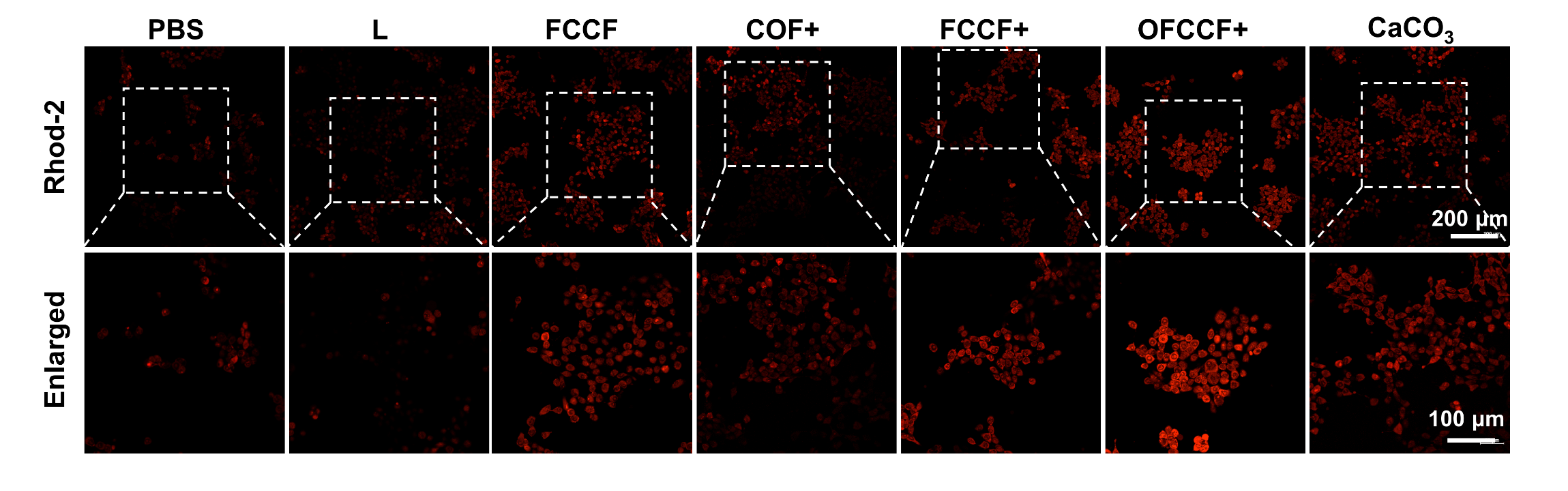


**Figure S13.** CLSM microimages of mitochondrial Ca^2+^ content in 4T1 cells.


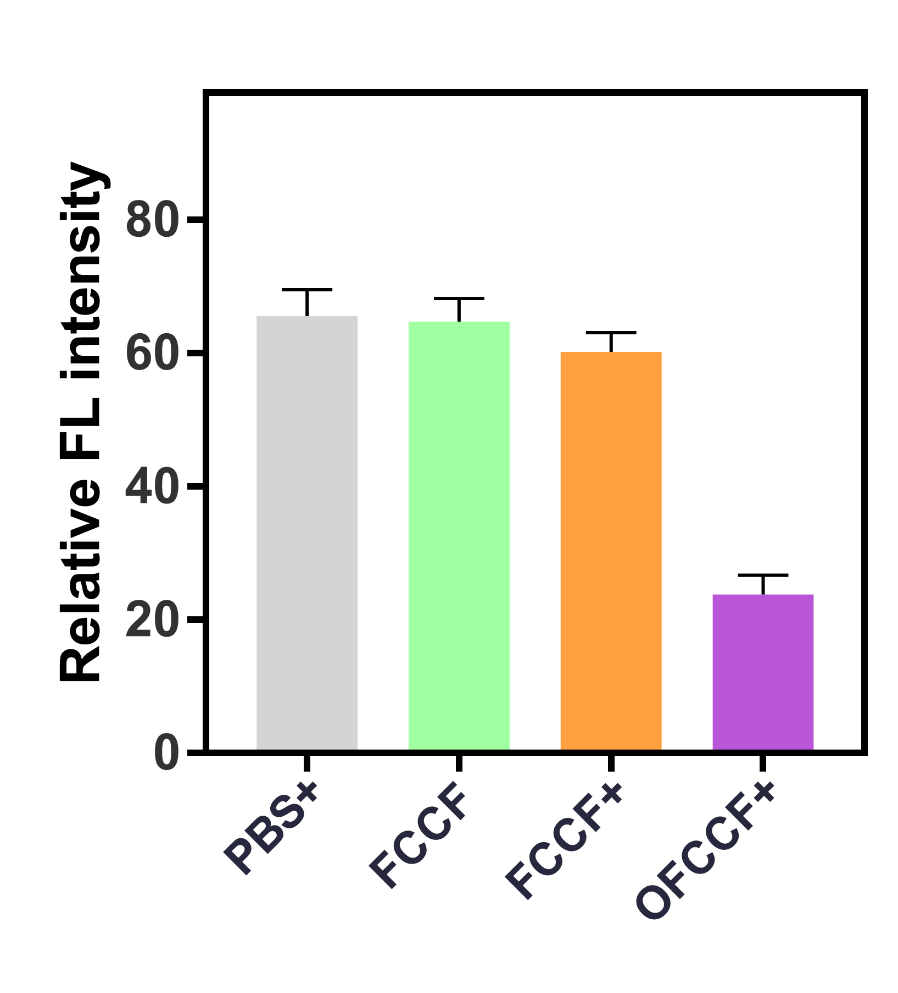

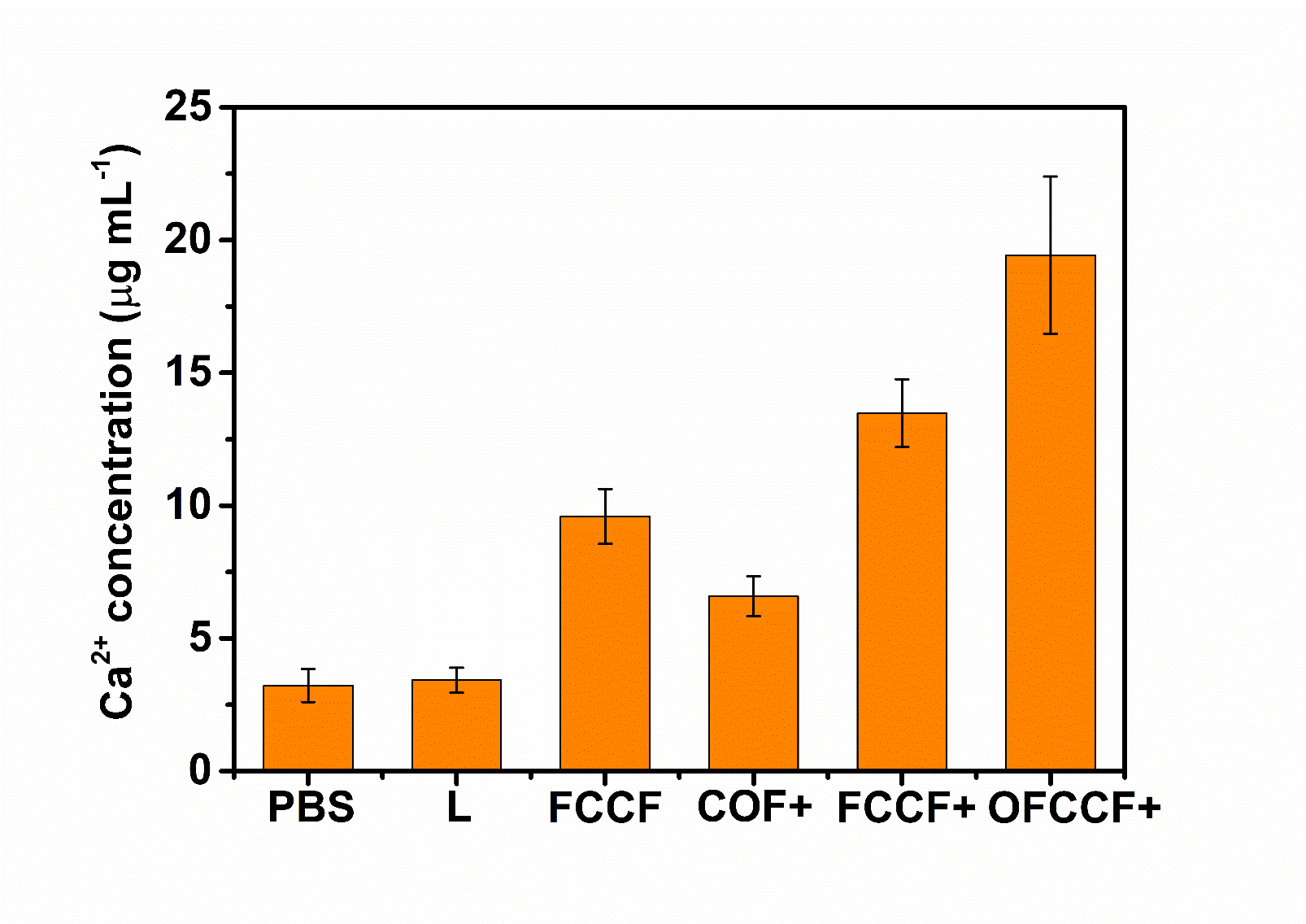
**Figure S14**. Intracellular Ca^2+^ concentration detection via Calcium Colorimetric assay in 4T1 cells.

**Figure S15**. Quantitative analysis of the intracellular O_2_ generation of 4T1 cells after various treatments based on the confocal images shown in Figure 3b by using imageJ, related to Figure 3b.


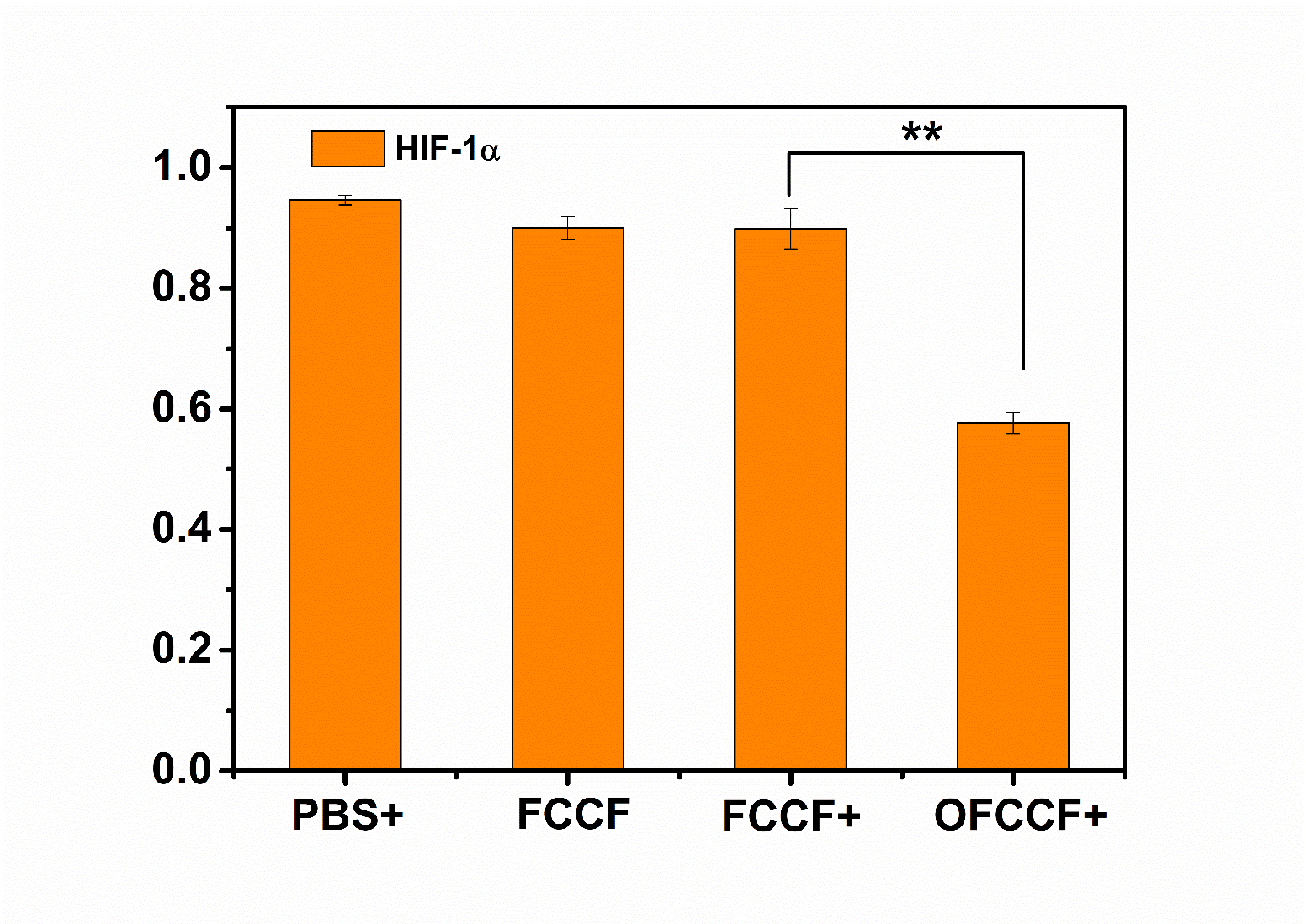
**Figure S16.** Quantitative analysis of HIF-1*α* protein expression, as the ratio of protein to β-actin from Western Blot results. Related to Figure 3c. *P* values were calculated by one-way analysis (**p*<0.05, ***p*<0.01, ****p*<0.001).

**
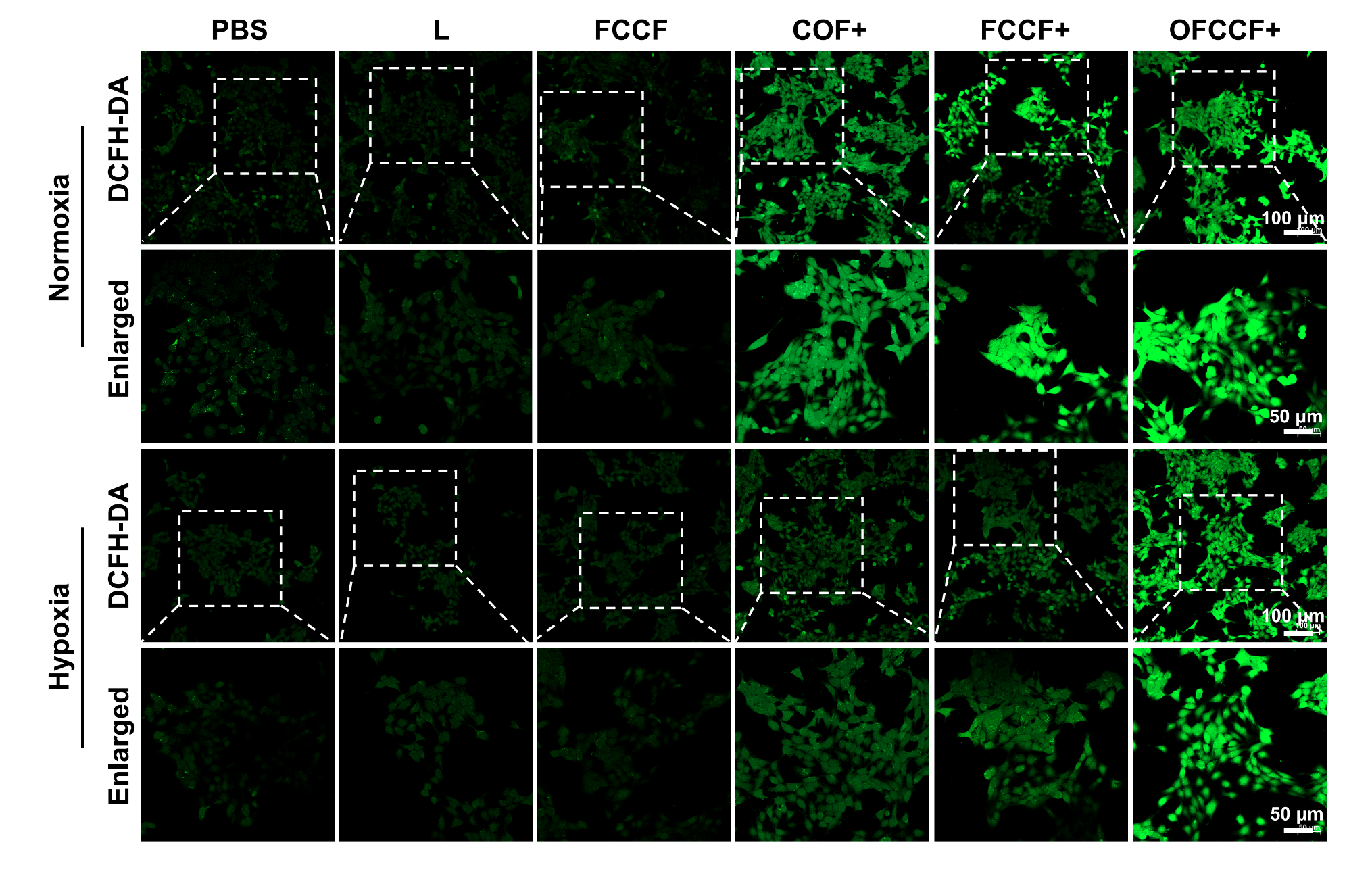
**

**Figure S17**. Intracellular ROS production of 4T1 cells under normoxic and hypoxic conditions after various treatments.


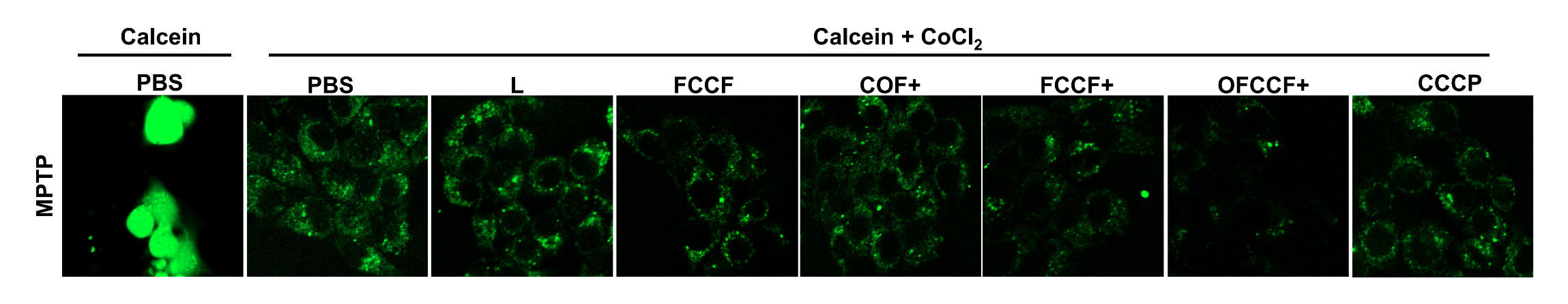


**Figure S18**. MPTP opening in mitochondria of 4T1 cells after different treatments.


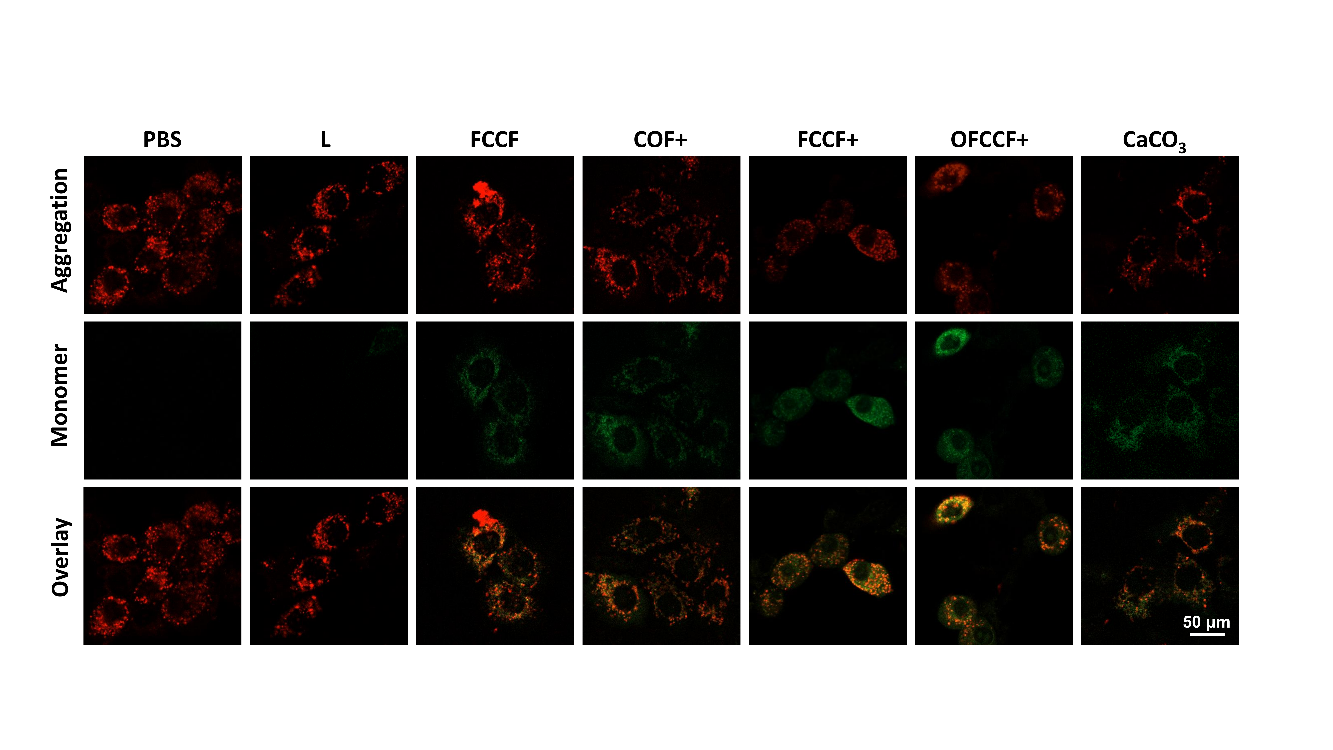


**Figure S19.** mitochondrial membrane potential images of 4T1 cells after different treatments.


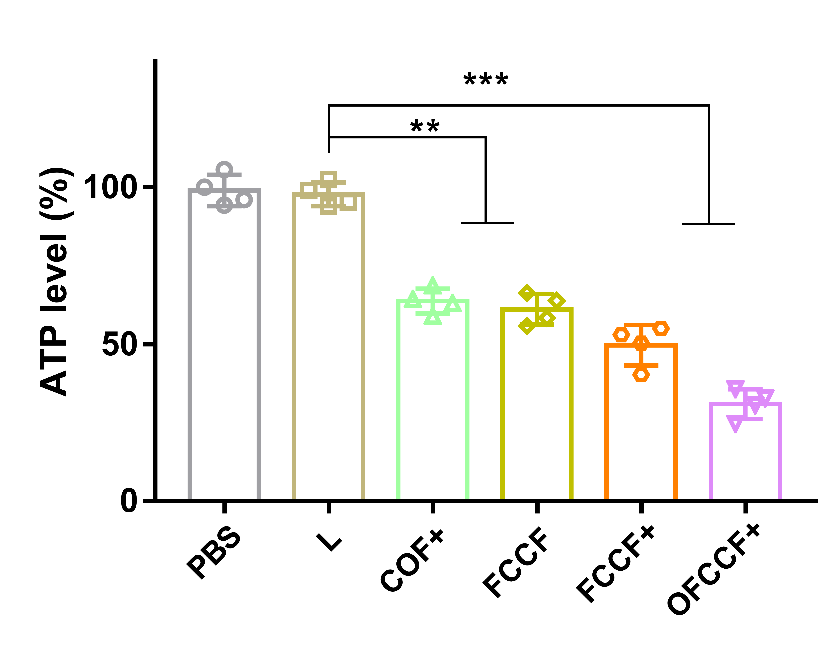


**Figure S20.** Changes of intracellular ATP contents of 4T1 cells after being treated with PBS, L, COF+, FCCF, FCCF+ and OFCCF+ for 24 h. *P* values were calculated by one-way analysis (**p*<0.05, ***p*<0.01, ****p*<0.001). Data were represented as mean ± SD (n = 4).


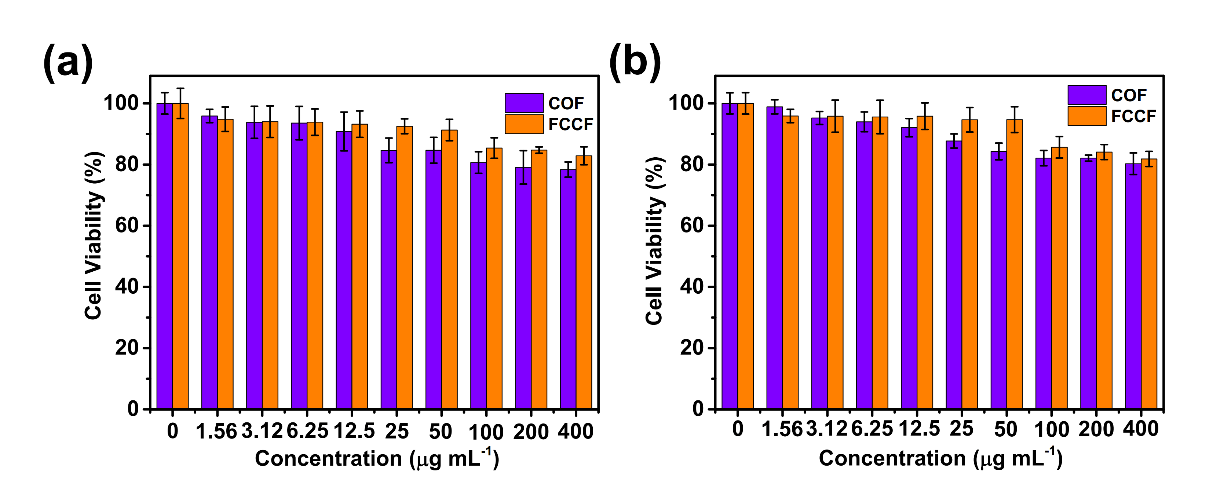


**Figure S21.** Relative viabilities of L929 cells treated by incubation with COF and FCCF at different concentration for (a) 24 h and (b) 48h. Data were represented as mean ± SD (n = 6).


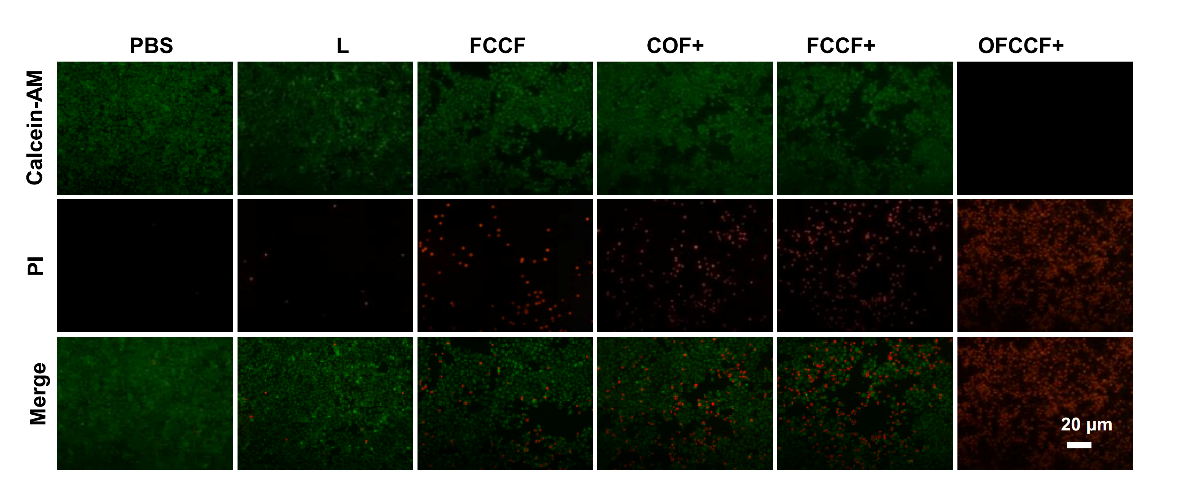


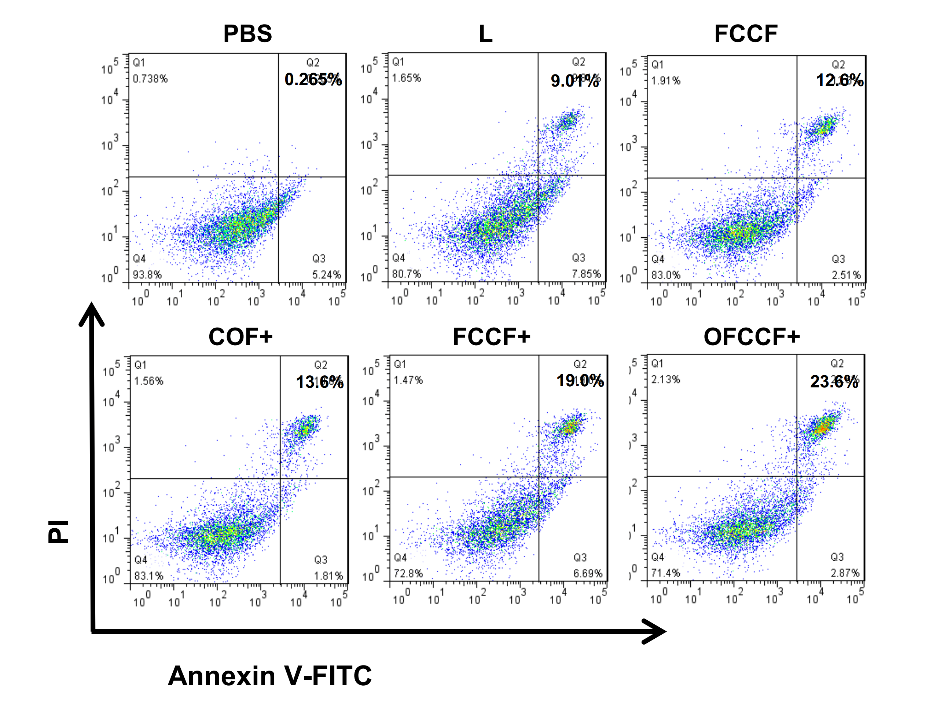
**Figure S22.** The images of 4T1 cells after various treatments and stained with calcein-AM (live cells: green) and PI (dead cell: red).

**Figure S23.** Quantitative analysis of Annexin V-FITC/PI co-stained 4T1 cells at 24 h after various treatments.


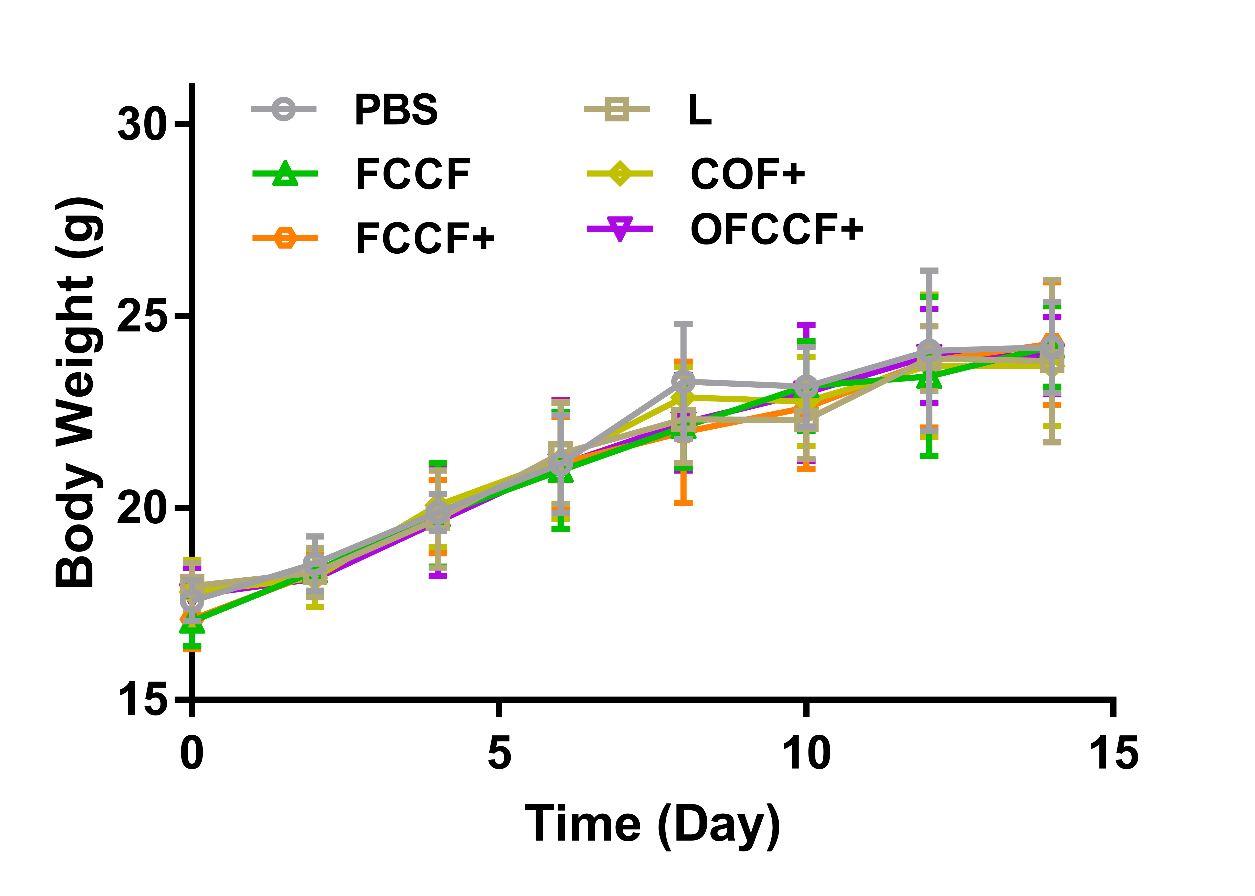


**Figure S24.** Changes of body weight of mice in different groups after treatment.


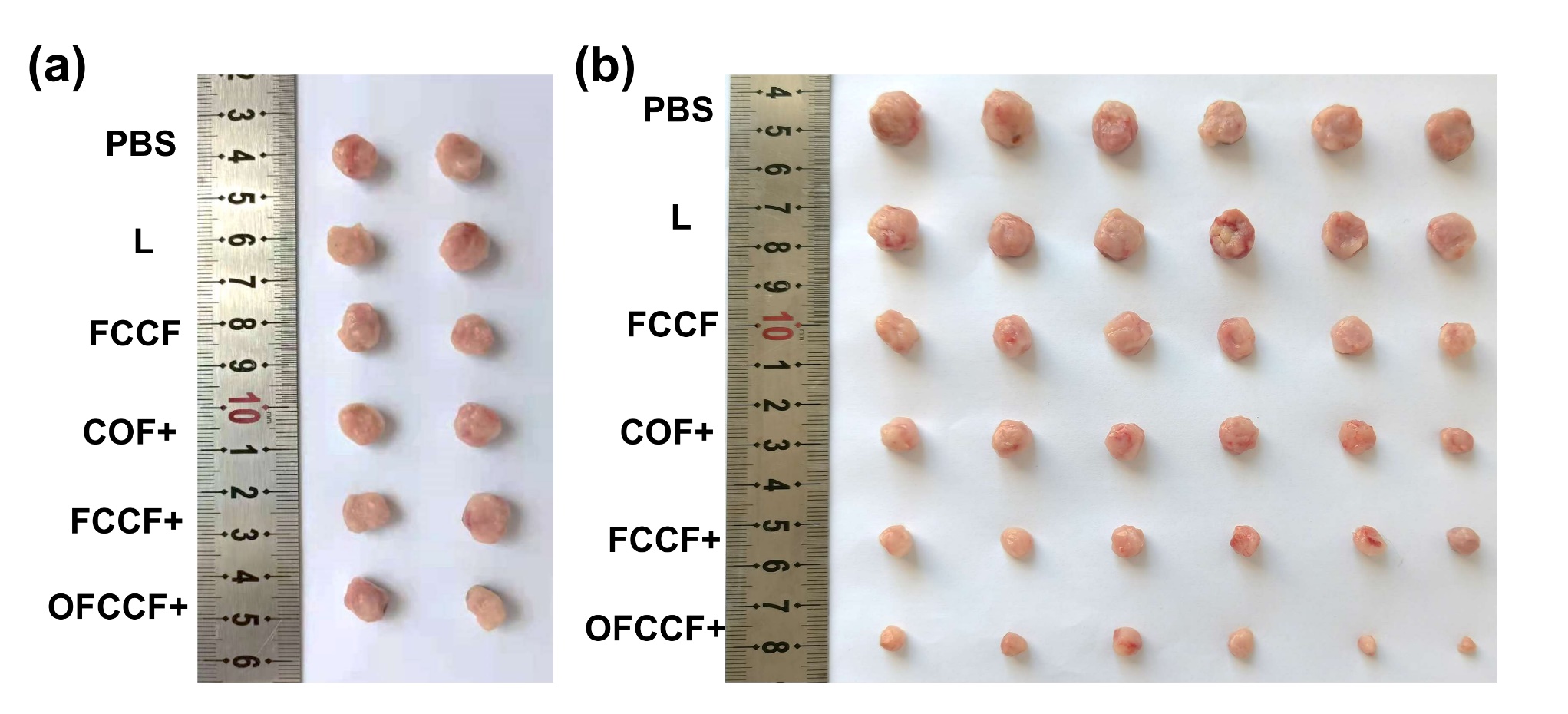


**Figure S25.** The tumor picture of mice before and after treatment in different groups (n = 8).

**
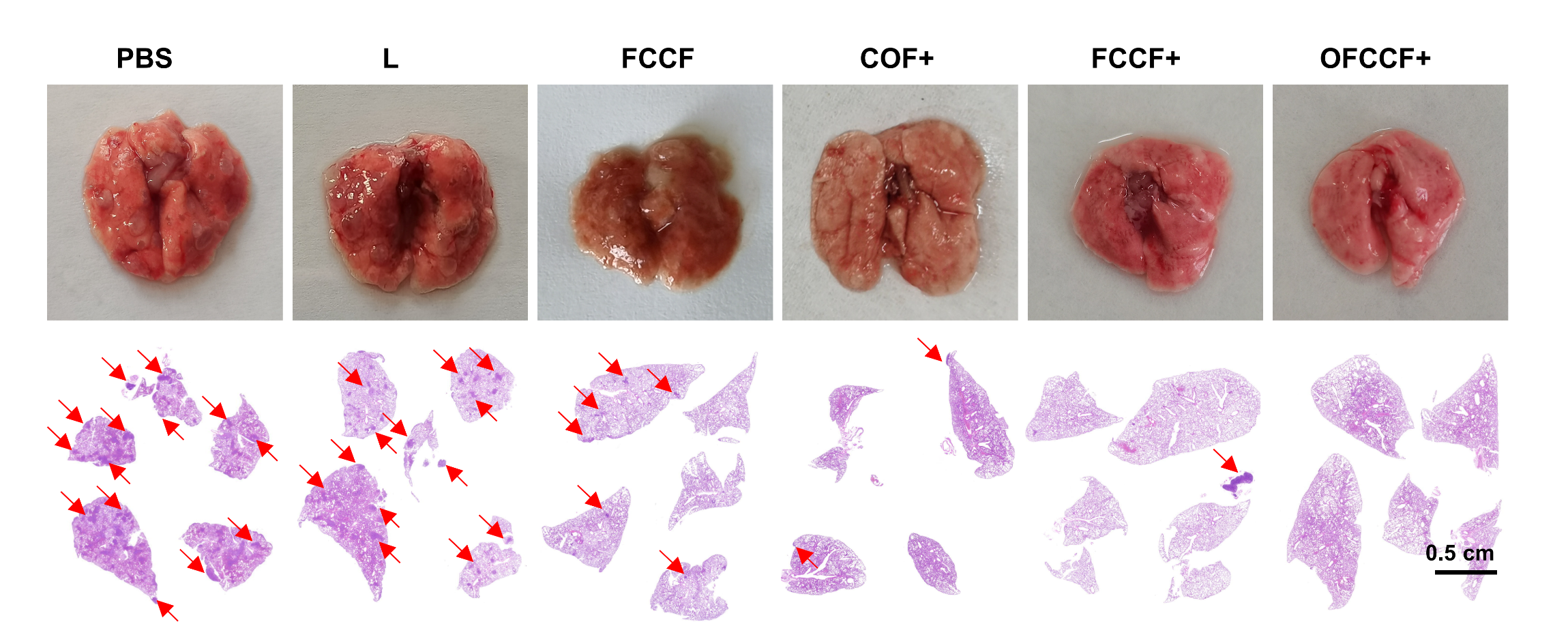
**

**Figure S26.** (a) The photos of lung at the end of various treatments. (b) H&E-stained lung sections at the end of various treatments. Red arrows indicate the sites of tumor metastasis.


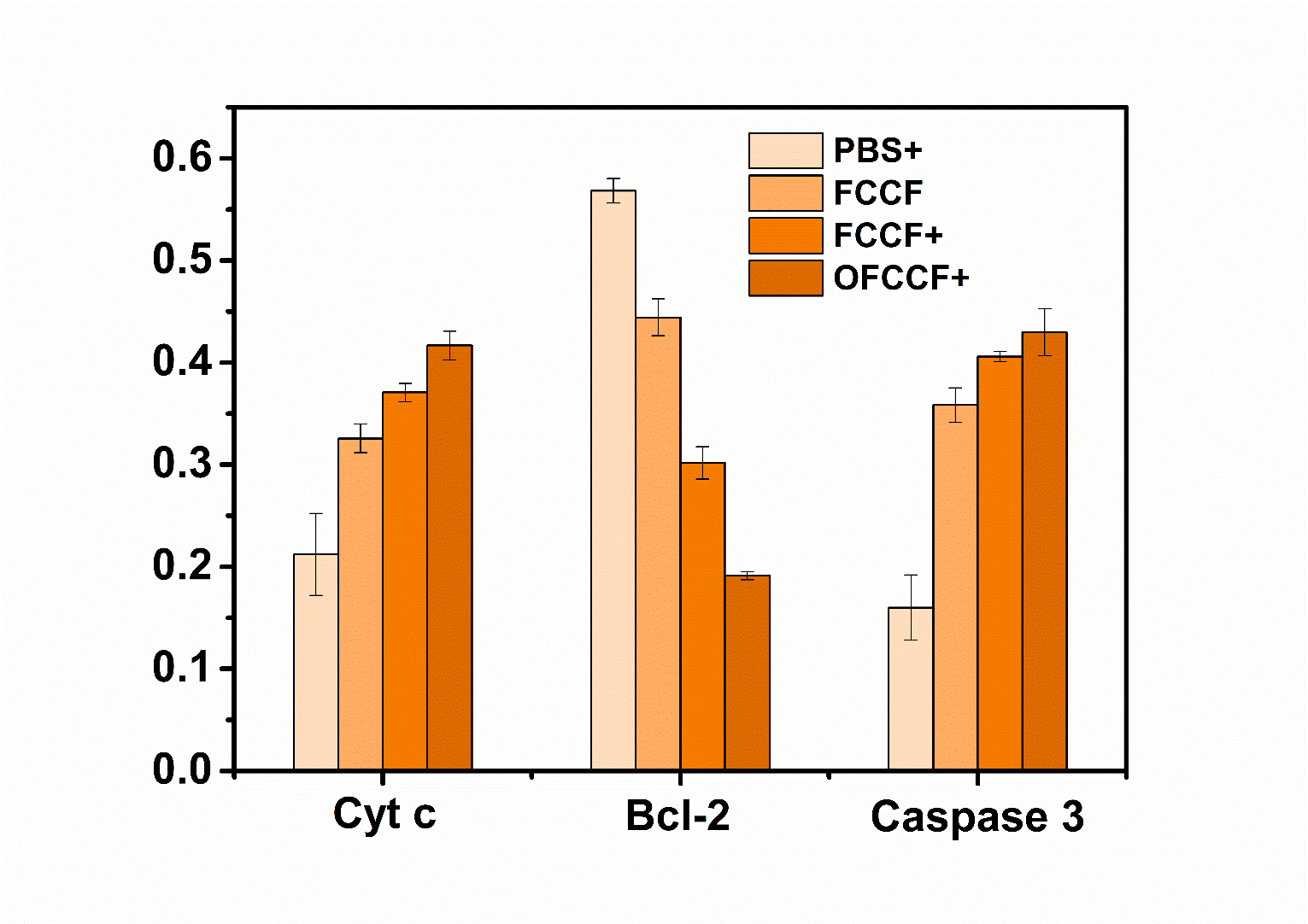


**Figure S27.** Quantitative analysis of caspase 3, Bcl-2 and Cyt c protein expression, as the ratio of protein to β-actin from Western Blot results. Related to Figure 6(f).


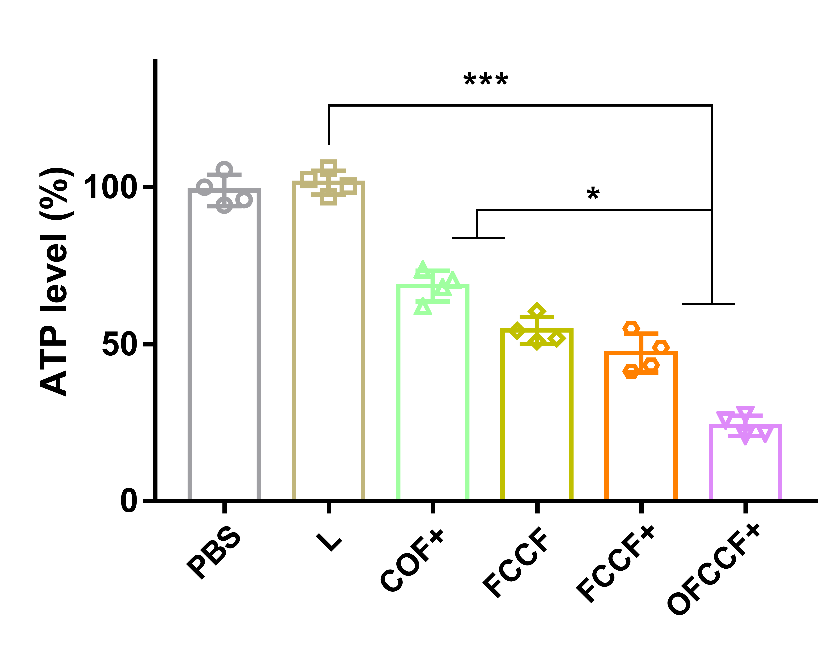


**Figure S28.** ATP contents of tumor after being treated with PBS, L, COF+, FCCF, FCCF+ and OFCCF+. *P* values were calculated by one-way analysis (**p*<0.05, ***p*<0.01, ****p*<0.001). Data are presented as mean ± SD (n = 4).


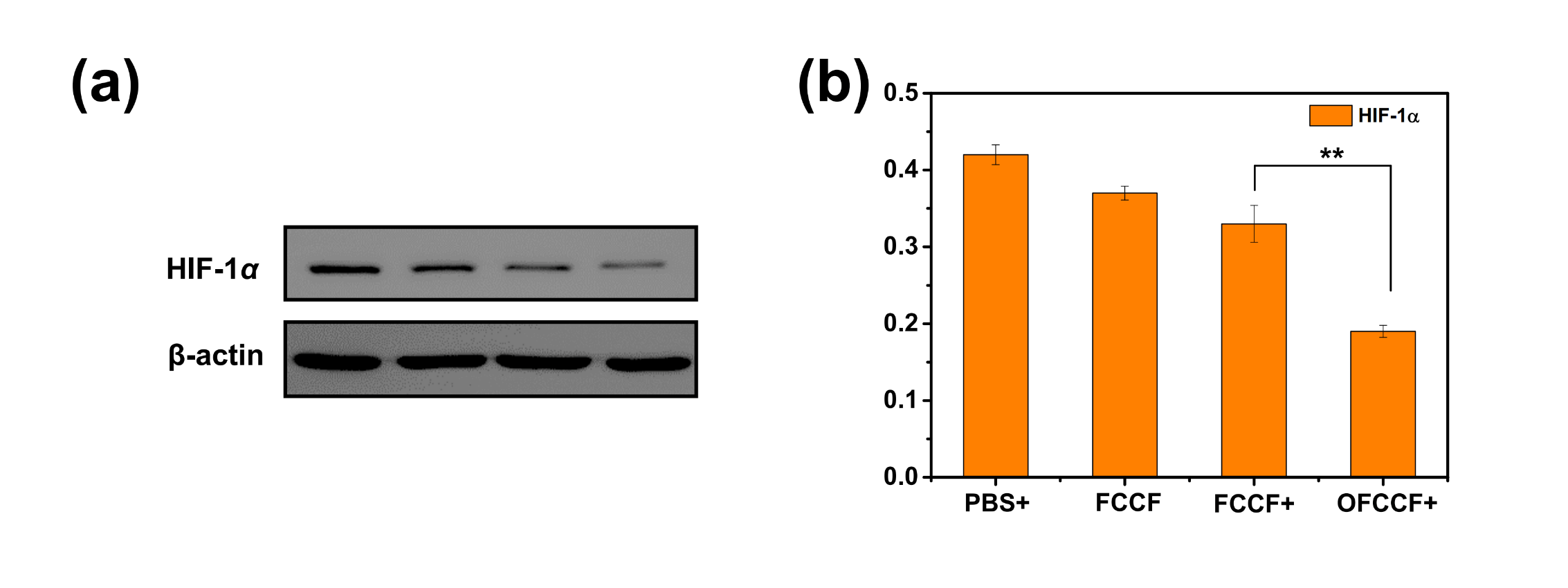
**Figure S29.** (a) The HIF-1*α* protein expression of tumor *via* western blotting analysis. (b) Quantitative analysis of HIF-1*α* protein expression, as the ratio of protein to β-actin from Western Blot results. *P* values were calculated by one-way analysis (**p*<0.05, ***p*<0.01, ****p*<0.001).


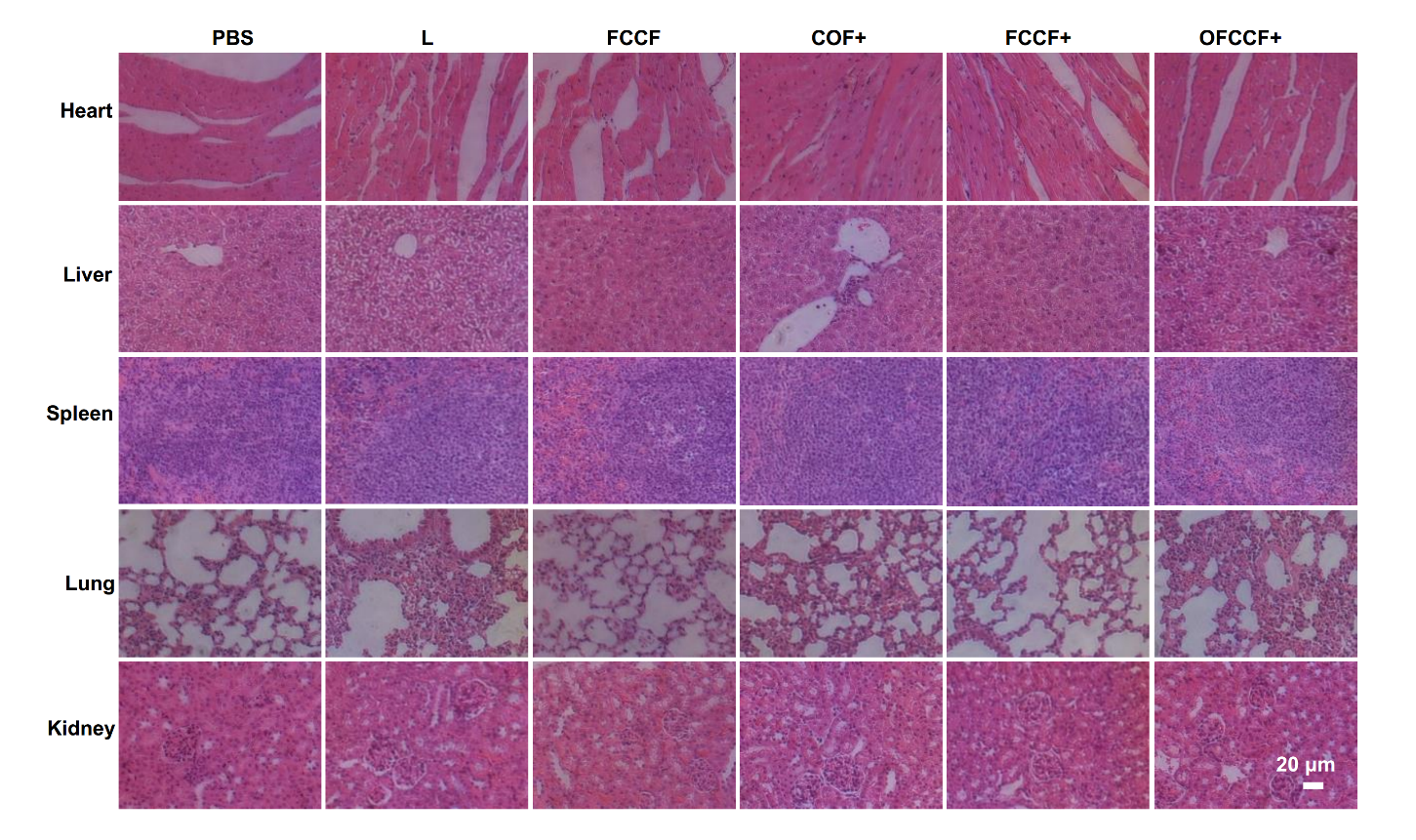


**Figure S30.** H&E stained images of the major organs, including heart, liver, spleen, lung, and kidney. Scale bar: 20 μm.


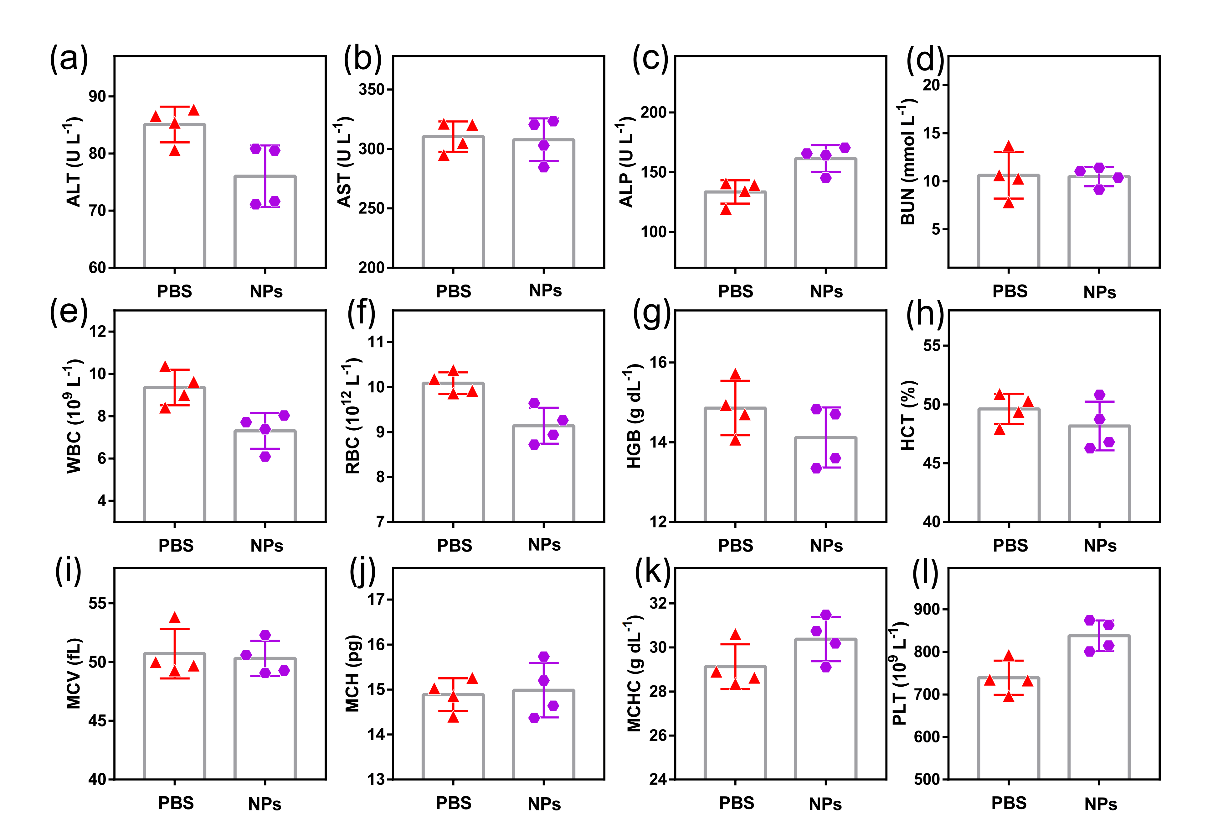
**Figure S31.** BALB/c mice were sacrificed after PBS and FCCF (NPs) treatment. Serum biochemistry data including Alanine aminotransferase (ALT), Aspartate aminotransferase (AST) and Alkaline phosphatase (ALP) as hepatic function indicators and Blood urea nitrogen (BUN) as renal function indicators were measured. Blood hematological counts: Blood levels of White blood cells (WBC), Red blood cells (RBC), Hemoglobin (HGB), Hematocrit (HCT), Mean corpuscular volume (MCV), Mean corpuscular hemoglobin (MCH), Mean corpuscular hemoglobin concentration (MCHC) and Blood platelet (PLT). Data are presented as mean ± SD (n = 4).
